# LncPEDS1-AS promotes UTUC resistance to lipid peroxidation by regulating PEDS1 expression via DDX23

**DOI:** 10.1038/s41419-025-08293-6

**Published:** 2025-12-08

**Authors:** Guanru Li, Erwei Zhang, Zhiyu Wang, Zhijin Zhang, Yuke Zhang, Shichen Di, Jingyi Lu, Shun Cao, Guoqing Xie, Yu Zhang, Keqiang Li

**Affiliations:** 1 Department of Urology, The First Affiliated Hospital of Zhengzhou University, Zhengzhou, China; 2 Academy of Medical Sciences, Zhengzhou University, Zhengzhou, Henan Province China; 3 Cancer Institute, School of Medicine, Tongji University, Shanghai, China; 4 Department of Urology, Shanghai Tenth People’s Hospital, Tongji University, Shanghai, China; 5 Department of Rheumatology and Immunology, Shanghai Children’s Medical Center, School of Medicine, Shanghai Jiao Tong University, Shanghai, China; 6 Department of Urology, Peking University First Hospital, Beijing, China

**Keywords:** Oncogenesis, Urological cancer

## Abstract

Upper tract urothelial carcinoma (UTUC) is a rare malignancy with a significantly poorer prognosis than bladder cancer (BC). One distinguishing feature of UTUC is its enhanced resistance to reactive oxygen species (ROS)-induced lipid peroxidation, a phenomenon closely associated with adverse clinical outcomes. However, the molecular mechanisms underlying this resistance remain largely unexplored. In this study, we identify LncPEDS1-AS, an ultra-long (>6900 nt) antisense lncRNA, as a key regulator of ROS resistance in UTUC. Mechanistically, LncPEDS1-AS interacts with the splicing factor DDX23, forming a nuclear RNA-protein complex that facilitates the splicing and maturation of PEDS1 pre-mRNA. PEDS1 encodes plasmanylethanolamine desaturase, which plays a protective role against lipid peroxidation. Based on these findings, we developed an antisense oligonucleotide (ASO) therapy strategy targeting LncPEDS1-AS, which effectively suppressed tumour growth and enhanced tumour cells’ ROS sensitivity both in vitro and in vivo. Moreover, our findings also highlight the distinctive molecular features and regulatory capacity of ultra-long antisense lncRNAs such as LncPEDS1-AS, which merit further comprehensive exploration in cancer biology.

**Figure:**
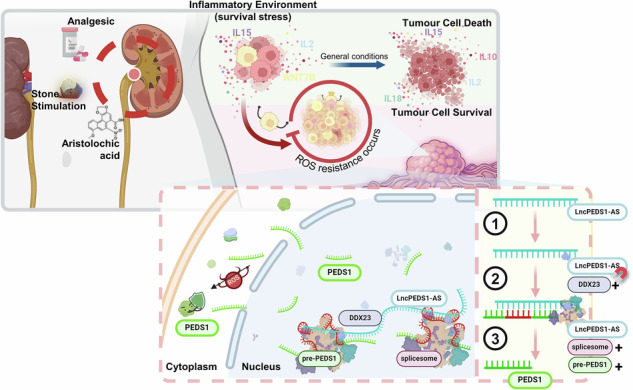
The LncPEDS1-AS-DDX23-PEDS1 axis is involved in resistance to lipid peroxidation and plays a critical role in the prognosis of UTUC (Created with BioRender.com).

The LncPEDS1-AS-DDX23-PEDS1 axis is involved in resistance to lipid peroxidation and plays a critical role in the prognosis of UTUC (Created with BioRender.com).

## Introduction

Upper tract urothelial carcinoma (UTUC) is a rare malignancy, characterised by its unique regional prevalence [1–3]. In the Pan-Asian region, the higher incidence of UTUC is closely associated with nephrotoxic herbal remedies, particularly those containing aristolochic acid, leading to chronic inflammation of the upper urinary tract [2, 3]. Despite its rarity, UTUC has garnered increasing attention due to its poorer prognosis compared to bladder cancer, another form of urothelial carcinoma [2, 4]. Clinically, UTUC often presents at advanced stages, exhibits a diminished responsiveness to chemotherapy, and lacks therapeutic modalities such as intravesical treatments, collectively contributing to UTUC’s poorer outcomes [5]. Consequently, a deeper understanding of the distinctive pathophysiology of UTUC is crucial.

Compared with bladder cancer, and from an aetiological perspective, UTUC is strongly associated with inflammation caused by factors such as aristolochic acid, analgesics, urinary calculi, and infection. During inflammatory responses, excessive reactive oxygen species (ROS) accumulation drives lipid peroxidation, representing a key contributor to cellular injury [1–3]. Arising from and persisting within an inflammatory environment, UTUC consequently exhibits a stronger resistance to the accumulation of ROS induced by stress conditions and is characterised by a poorer prognosis [4, 6].

Increasing evidence underscores the role of long non-coding RNAs (lncRNAs) in numerous biological processes. Although lncRNAs do not code for proteins, they modulate gene expression, subsequently influencing cellular phenotypes through various mechanisms [7–10]. The mechanisms through which lncRNAs can regulate mRNA expression are either by serving as molecular sponges that bind miRNAs or through direct interactions with proteins. This second mechanism can be further divided into two subtypes. One subtype involves lncRNAs acting as decoys, attracting RNA-degrading proteins and thereby protecting mRNAs from nucleolytic degradation. The other subtype involves lncRNA-binding proteins that promote either DNA transcription or RNA maturation [11, 12].

Among the diverse mechanisms by which lncRNAs exert their regulatory functions, increasing attention has been directed toward their involvement in RNA splicing [9, 13, 14]. RNA splicing is a critical post-transcriptional process that precisely removes introns from precursor mRNA and joins exons to form mature transcripts. Aberrations in this process have been shown to directly affect tumour progression and therapeutic responses [9, 13, 14]. Members of the RNA helicase family serve as key splicing regulators, among which DEAD-box RNA helicase 23 (DDX23) plays a pivotal role. As a component of the U5 small nuclear ribonucleoprotein (U5 snRNP) complex, DDX23 is involved in spliceosome assembly and pre-mRNA processing, thereby contributing to the maturation and stability of mRNA transcripts [15, 16].

Antisense lncRNAs, as one particular type of lncRNA, partially overlap with the exons of their associated sense genes. This unique structural and positional attribute often leads to a closer interaction and more potent influence on their associated sense genes [17, 18].

In light of this, our study, based on high-throughput sequencing, identifies a novel antisense lncRNA, LncPEDS1-AS, which is closely associated with UTUC. As the sense mRNA of LncPEDS1-AS is PEDS1, it encodes the protein plasmanylethanolamine desaturase, which is responsible for transforming alkyl ether lipids into plasmalogens [19, 20]. This transformation is pivotal in protecting polyunsaturated ether phospholipids (PUFA-PLs) against lipid peroxidation, consequently reducing the sensitivity of cells to lipid peroxidation damage induced by saturated fatty acids (SFA) and PUFA-ePLs [19–21].

Our research provides a detailed explanation of the regulatory effect of LncPEDS1-AS on PEDS1 and elucidates the role of this regulatory axis in resisting lipid peroxidation in UTUC. This insight offers potential for specific therapeutic strategies tailored to UTUC.

## Results

### High expression of LncPEDS1-AS in UTUC is closely related to worse survival

**Fig. 1:**
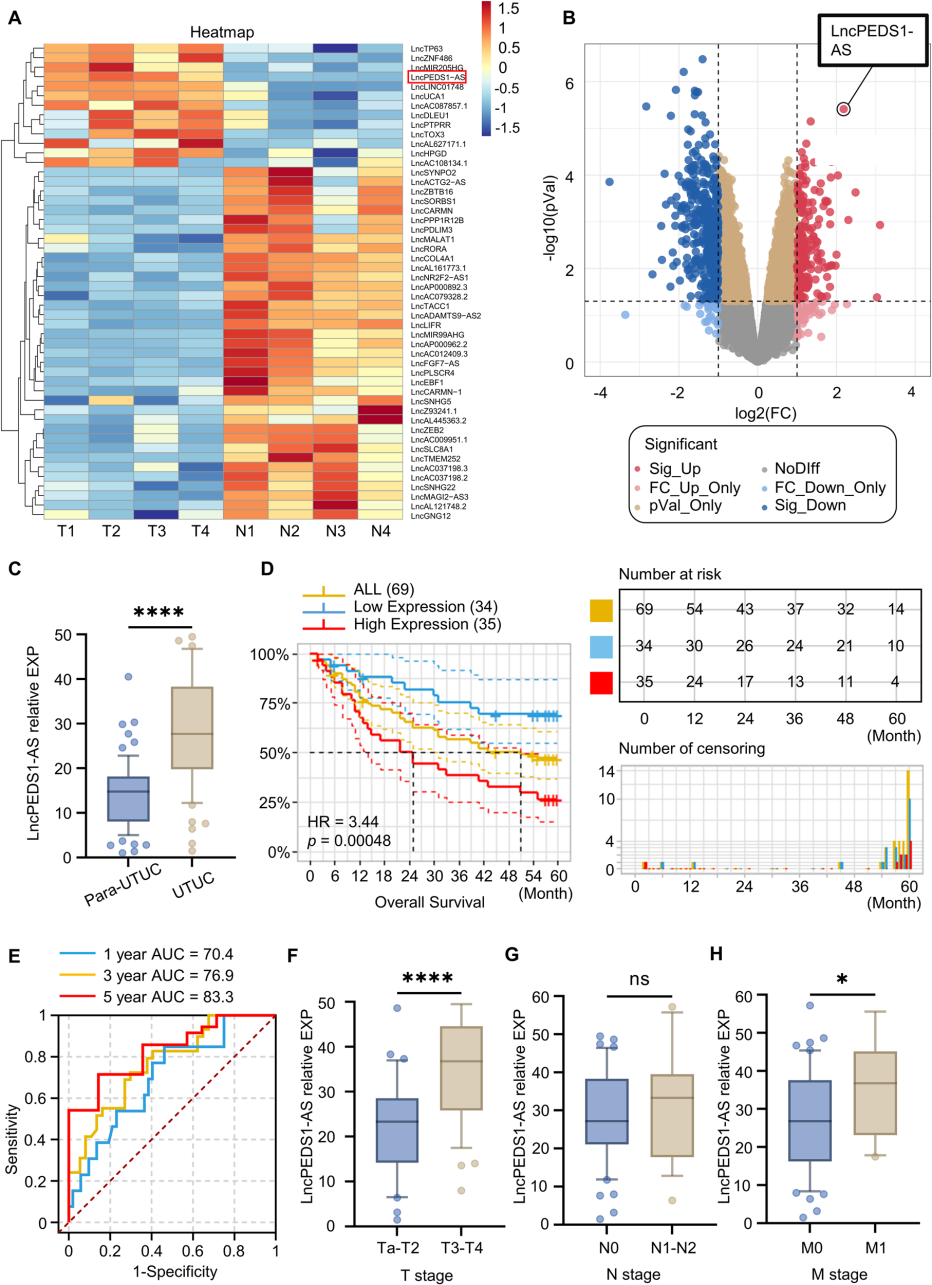
LncPEDS1-AS is highly expressed in UTUC and is associated with poor prognosis in UTUC patients. **A**, **B** Heatmap and volcano plot showing differentially expressed lncRNAs in tumour and adjacent normal tissues of UTUC patients based on high-throughput sequencing (*n* = 4). **C** Differential expression of LncPEDS1-AS in tumour and adjacent normal tissues of UTUC patients (*n* = 69). **D** Correlation between LncPEDS1-AS expression and prognosis in UTUC patients (*n* = 69). **E** ROC analysis of LncPEDS1-AS expression in UTUC tumour tissues for predicting 1-year, 3-year, and 5-year prognosis (*n* = 69). **F**–**H** Relationship between LncPEDS1-AS expression and TNM staging in UTUC patients (*n* = 69). (*p* > 0.05 as ns, *p* ≤ 0.05 as *, *p* ≤ 0.0001 as ****).

To investigate the molecular mechanisms of UTUC and the role of non-coding RNAs, we selected four tumour specimens with confirmed inflammation-related aetiology, along with their adjacent normal tissues, from a UTUC sample repository for transcriptome sequencing. The sequencing results revealed that the antisense lncRNA, LncPEDS1-AS, exhibited significant differential expression between tumour and adjacent normal tissues (Figs. 1A, B and S1A). Moreover, its sense mRNA, PEDS1, was found to be closely associated with resistance to lipid peroxidation and the clearance of ROS. Therefore, we hypothesise that LncPEDS1-AS may play a role in intracellular resistance to lipid peroxidation and may be associated with the poor prognosis of UTUC driven by ROS resistance.

To clarify the relationship between LncPEDS1-AS expression and UTUC prognosis, we first validated the differential expression of LncPEDS1-AS in 69 pairs of UTUC tumour and adjacent tissue samples. Consistent with our sequencing results, LncPEDS1-AS expression was higher in tumour tissues. While this difference was not significant in urothelial carcinoma of the bladder (UCB) (Figs. 1C and S1B–D).

Then we conducted survival analysis on 69 tumour samples based on the expression levels of LncPEDS1-AS. We found that high expression of LncPEDS1-AS is closely related to worse survival. ROC analysis also revealed that LncPEDS1-AS could serve as an independent risk factor in 1-year, 3-year, and 5-year survival prediction, with a high AUC value (Fig. 1D, E). Additionally, we further analysed the relationship between the expression level of LncPEDS1-AS and the TNM stage of UTUC tumours. The results showed a positive correlation between the expression level of LncPEDS1-AS and the T stage (Fig. 1F–H).

### The expression level of LncPEDS1-AS affects cell survival under ROS stimulation

**Fig. 2:**
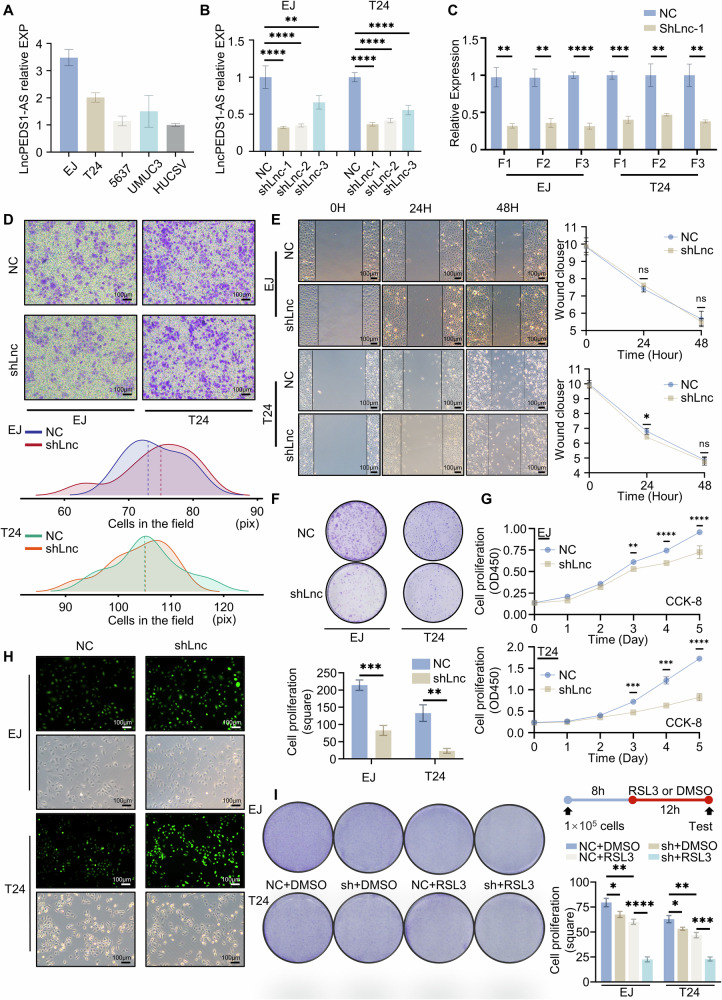
LncPEDS1-AS affects the survival capacity of urothelial carcinoma cells. **A** Expression levels of LncPEDS1-AS in different urothelial carcinoma cell lines. **B** Effects of LncPEDS1-AS knockdown at different target sites on its expression. **C** Changes in the expression of different LncPEDS1-AS fragments after knockdown in EJ and T24 cell lines. **D** Transwell assay comparing control and LncPEDS1-AS knockdown groups. Scale bar, 100 μm. **E** Wound-healing assay comparing control and LncPEDS1-AS knockdown groups. Scale bar, 100 μm. **F**, **G** Colony formation assay and CCK8 assay comparing control and LncPEDS1-AS knockdown groups. **H** Differences in ROS accumulation between control and LncPEDS1-AS knockdown groups. Scale bar, 100 μm. **I** Sensitivity to RSL3 (6 µM) in the LncPEDS1-AS knockdown group and the control group. (Data are shown as the mean ± SD, *n* = 3. *p* > 0.05 as ns, *p* ≤ 0.05 as *, *p* ≤ 0.01 as **, *p* ≤ 0.001 as ***, *p* ≤ 0.0001 as ****).

To further study the functions of LncPEDS1-AS, we first tested the expression of LncPEDS1-AS in the cell line of urothelial carcinoma. The results showed that the expression of LncPEDS1-AS was high in the EJ and T24. Simultaneously, it was found that the expression of LncPEDS1-AS in the urothelial carcinoma cell line was higher than in the normal urothelial cell line HUCSV (Fig. 2A). Therefore, we performed lentiviral knockdown of LncPEDS1-AS in the EJ and T24 cell lines, respectively. As this lncRNA is quite long (approximately 7000nt) (Data 1), we designed three different knockdown sites, located at the front, middle, and end of this lncRNA, respectively named as sh1, sh2, and sh3 (Fig. S1E). After the knockdown experiment, we found that all three knockdown sites had a significant knockdown effect on LncPEDS1-AS. Among them, sh1 had the best knockdown effect in both EJ and T24 (Fig. 2B). Therefore, we chose the sh1 knockdown group of EJ and T24 for subsequent experiments. Additionally, as some studies have suggested that longer lncRNAs are more likely to generate truncated fragments that may give rise to new lncRNAs following knockdown, we conducted a more rigorous analysis to account for this possibility. Specifically, we used primers targeting the front, middle, and end sections of LncPEDS1-AS to test the knockdown effect in the two cell lines. Our results indicated a significant decrease in expression across all three sections, confirming the effective knockdown of LncPEDS1-AS without the formation of detectable by-products (Fig. 2C).

To investigate the relationship between LncPEDS1-AS expression and tumour cell migration, invasion, and survival, we first performed transwell assays, scratch assays, and colony formation assays. The results demonstrated that LncPEDS1-AS expression was closely associated with cell survival but showed no significant correlation with either migration or invasion (Fig. 2D–F). We further validated the association between LncPEDS1-AS expression and cell survival using the CCK8 assay. The cell survival ability of the LncPEDS1-AS knockdown group was significantly lower than that of the control group (Fig. 2G).

Given that the poor prognosis of UTUC is associated with ROS resistance and that the sense strand of LncPEDS1-AS, PEDS1, is known to play a role in ROS resistance [19, 20], we hypothesised that LncPEDS1-AS may influence cell survival by modulating ROS sensitivity. To test this hypothesis, we examined the accumulation of ROS in cell lines following LncPEDS1-AS knockdown. The results revealed a significant increase in intracellular ROS levels in the LncPEDS1-AS knockdown group compared to the control group (Fig. 2H). Additionally, experiments assessing sensitivity to ROS accumulation indicated that knockdown of LncPEDS1-AS significantly increased cellular sensitivity to RSL3-induced ROS accumulation (Figs. 2I and S1F).

### LncPEDS1-AS mediates lipid peroxidation in urothelial carcinoma cells through PEDS1

**Fig. 3:**
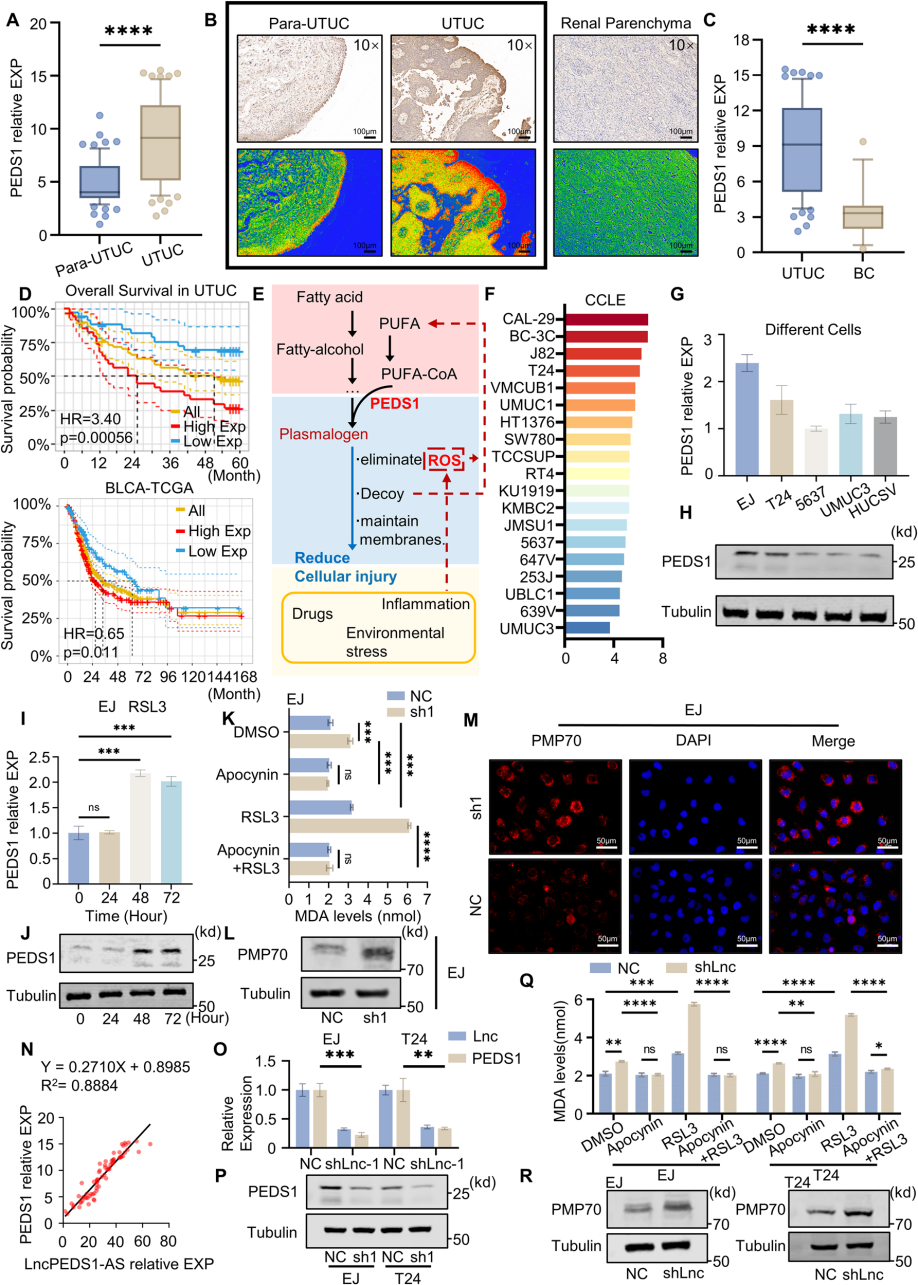
LncPEDS1-AS regulates lipid peroxidation through PEDS1. **A**, **B** Differences in PEDS1 expression between tumour tissues and adjacent normal tissues in UTUC patients (*n* = 69). Scale bar, 100 μm. **C** Differences in PEDS1 expression in tissue samples of UTUC and bladder urothelial carcinoma (BC *n* = 34, UTUC *n* = 69). **D** The impact of PEDS1 expression on the prognosis of UTUC and bladder cancer patients. **E** Schematic illustration of the mechanism by which PEDS1 influences ROS accumulation and lipid peroxidation. **F**–**H** PEDS1 expression levels in different urothelial carcinoma cell lines. **I**, **J** The effect of different treatment durations with RSL3 on PEDS1 expression. **K** MDA content in control and PEDS1 knockdown cells after treatment with Apocynin (10 µM) and RSL3 (1 µM). **L**, **M** Expression levels of PMP70 in control and PEDS1 knockdown EJ cells. Scale bar, 50 μm. **N** Correlation analysis of LncPEDS1-AS and PEDS1 expression in UTUC tumour samples (*n* = 69). **O**, **P** PEDS1 expression levels in control and LncPEDS1-AS knockdown cells. **Q** MDA content in control and LncPEDS1-AS knockdown cells after treatment with Apocynin (10 µM) and RSL3 (1 µM). **R** Expression levels of PMP70 in control and LncPEDS1-AS knockdown cells. (Data are shown as the mean ± SD, unless otherwise specified, *n* = 3. *p* > 0.05 as ns, *p* ≤ 0.05 as *, *p* ≤ 0.01 as **, *p* ≤ 0.001 as ***, *p* ≤ 0.0001 as ****).

The lncRNAs do not encode proteins; however, they can directly and indirectly affect cellular phenotypes through various mechanisms, primarily by modulating gene expression [11, 12]. As a subclass of lncRNAs, antisense lncRNAs often function by modulating their corresponding sense mRNAs [17]. The sense strand mRNA of LncPEDS1-AS is PEDS1, which is highly expressed in most types of tumours. Through RT-qPCR experiments and bioinformatic analysis based on the TCGA database, we found that PEDS1 has significant differences in two types of urothelial carcinoma and is related to the worse survival in both types of urothelial carcinoma, particularly in UTUC (Figs. 3A–D and S2A–C). PEDS1 encodes plasmanylethanolamine desaturase, which can transform alkyl ether lipids into plasmalogens. It protects PUFA-PLs from lipid peroxidation to reduce the sensitivity of cells to lipid peroxidation damage caused by SFA and polyunsaturated ether phospholipids (PUFA-ePLs). Lipid peroxidation-induced cell death is the main form of ROS-induced cell death. For UTUC, which is closely related to ROS, high expression of PEDS1 may play a critical role in diminishing tumour sensitivity to ROS-induced cell death, leading to a poor prognosis (Fig. 3E) [19–21].

To confirm that the biological process by which PEDS1 inhibits lipid peroxidation-induced cell death is also present in UTUC, we selected the urothelial carcinoma cell lines EJ and T24 for further investigation due to their high PEDS1 expression levels (Fig. 3F–H) [18]. First, we induced ROS accumulation in these cells using RSL3 and observed that PEDS1 levels significantly increased with prolonged RSL3 exposure, consistent with previous research (Figs. 3I, J and S2D, E) [20]. Next, we knocked down the PEDS1 gene (Fig. S2F–I). In the PEDS1 knockdown group, the levels of malondialdehyde (MDA), a terminal product of lipid peroxidation, were significantly increased. Notably, this increase could be mitigated by the ROS scavenger Apocynin. Similarly, the levels of PMP70, a peroxisomal transporter protein involved in lipid peroxidation, were markedly increased in the PEDS1 knockdown group (Figs. 3K–M and S2J–N). We further observed that silencing PEDS1 markedly decreased the antioxidant effectors NRF2, GPX4, FSP1 and SOD1, indicating impairment of the core antioxidant pathway, whereas PEDS1 overexpression produced only a modest increase in these protective proteins. This pattern is consistent with previous reports that PEDS1 operates within a negative-feedback circuit to resist lipid peroxidation and functions as a threshold-dependent requisite factor rather than a dose-responsive amplifier (Fig. S3A–H) [20]. These findings demonstrate that PEDS1 continues to reduce cellular sensitivity to ROS-induced lipid peroxidation and thereby enhances tumour cell survival in urothelial carcinoma.

To confirm whether the effect of LncPEDS1-AS on the phenotypes of upper urothelial carcinoma cells is mediated via PEDS1, we examined the correlation between LncPEDS1-AS and PEDS1 expression in 69 UTUC tumour samples. A significant positive correlation was observed between LncPEDS1-AS and PEDS1 (Fig. 3N). More importantly, in the LncPEDS1-AS knockdown group, PEDS1 mRNA levels were significantly reduced, and lipid peroxidation sensitivity was increased. Notably, this increased sensitivity to lipid peroxidation could also be mitigated by the addition of Apocynin (Fig. 3O–R). These findings indicate that LncPEDS1-AS exerts its effects on tumour cell phenotypes through PEDS1.

### Investigation of the mechanism of LncPEDS1-AS modulating PEDS1

**Fig. 4:**
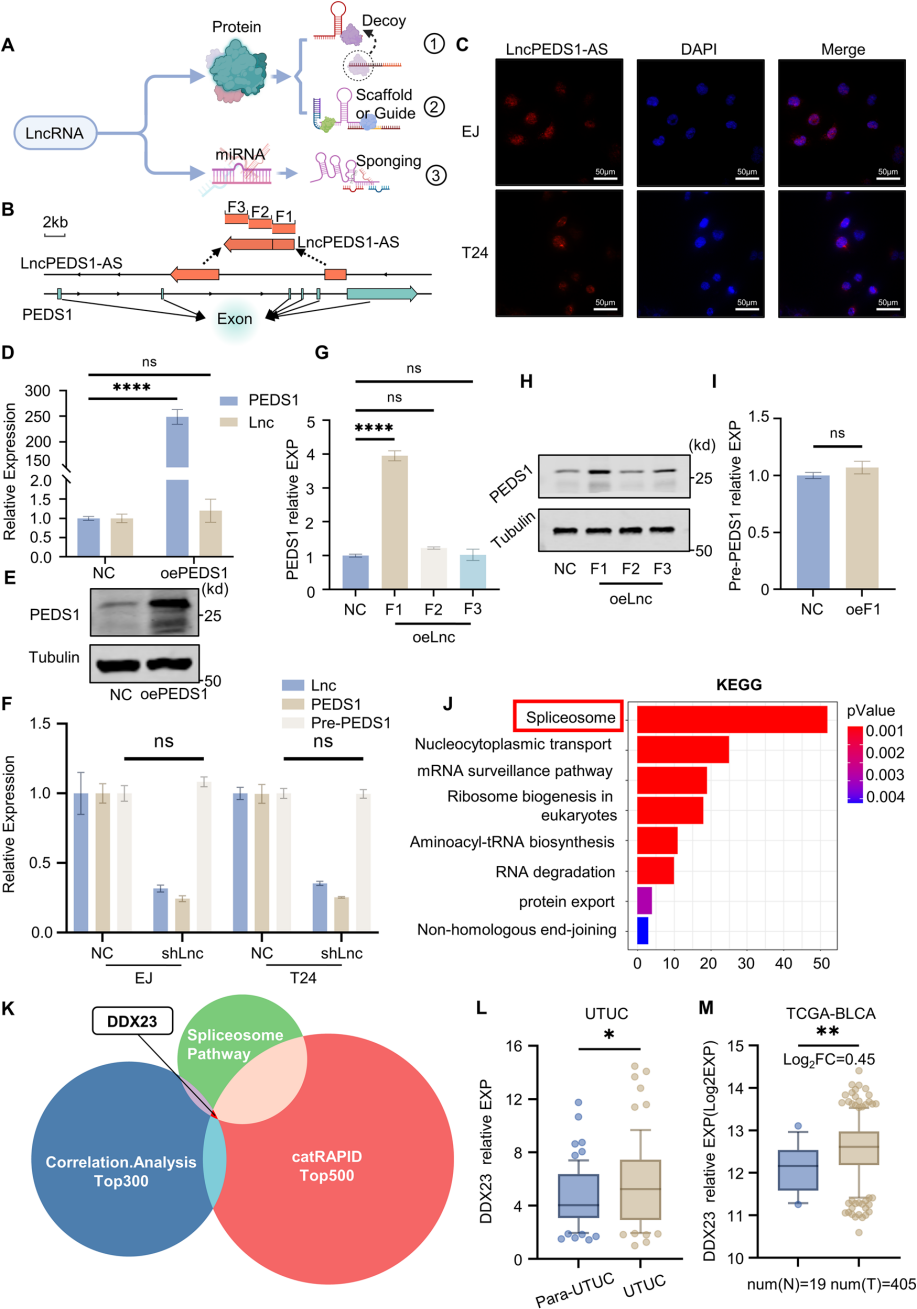
Mechanistic investigation of how LncPEDS1-AS regulates PEDS1. **A** Schematic diagram of the mode of action of LncRNAs (Created with BioRender.com). **B** Gene structure of LncPEDS1-AS and PEDS1. **C** Fluorescence in situ hybridisation (FISH) showing the subcellular localisation of LncPEDS1-AS. Scale bar, 50 μm. **D**, **E** Expression levels of LncPEDS1-AS in control and PEDS1 overexpression groups. **F** Precursor PEDS1 and mature PEDS1 levels in control and LncPEDS1-AS knockdown groups. **G**, **H** Changes in PEDS1 expression after overexpressing different fragments of LncPEDS1-AS. **I** Levels of precursor PEDS1 in control and overexpression groups of the F1 fragment of LncPEDS1-AS. **J** Enrichment analysis of LncPEDS1-AS-interacting proteins. **K** Venn diagram of LncPEDS1-AS-interacting proteins, spliceosome pathway genes, and PEDS1 co-expressed genes. **L**, **M** Differential expression of DDX23 in tumour and adjacent normal tissues of UTUC and bladder cancer samples (UTUC *n* = 69, TCGA-BLCA *n* = 424(N19, T405)). (Data are shown as the mean ± SD, unless otherwise specified, *n* = 3. *p* > 0.05 as ns, *p* ≤ 0.05 as *, *p* ≤ 0.01 as **, *p* ≤ 0.0001 as ****).

Although we have identified a close association between the high expression of PEDS1 in UTUC and LncPEDS1-AS, the precise regulatory mechanism underlying this relationship remains unclear. The mechanisms by which antisense lncRNAs regulate mRNA expression can broadly be categorised into two classes. The first involves the ability of antisense lncRNAs, as a subclass of lncRNAs, to influence mRNA expression through a sponging effect by binding to miRNAs. The second category pertains to the regulation of mRNA expression through interactions with proteins (Fig. 4A) [11, 12, 17].

LncPEDS1-AS is over 6900 nucleotides in length, making it longer than most lncRNAs. It is transcribed from the antisense strand of at least two introns (Fig. 4B). To investigate the mechanism by which LncPEDS1-AS regulates PEDS1, we first utilised FISH probes to determine the subcellular localisation of this lncRNA (Fig. 4C). The results revealed that LncPEDS1-AS is predominantly located in the cell nucleus. This finding aligns with our hypothesis, as the ability of RNA to exit the nucleus is closely associated with its length. Furthermore, since the miRNAs sponging process occurs in the cytoplasm, the nuclear localisation of LncPEDS1-AS suggests that it does not regulate PEDS1 expression through miRNAs sponging mechanism.

Therefore, LncPEDS1-AS regulates the expression of PEDS1 through its interaction with proteins. To further investigate the underlying regulatory mechanism, we conducted three sets of experiments: (A) In the first experiment, results showed that overexpression of PEDS1 did not lead to an upregulation of LncPEDS1-AS expression (Fig. 4D, E). (B) In the second experiment, we compared the expression levels of PEDS1 mRNA and pre-mRNA between the LncPEDS1-AS knockdown group and the control group. While PEDS1 mRNA expression was significantly reduced in the knockdown group, the expression level of PEDS1 pre-mRNA remained unchanged (Fig. 4F). (C) In the third experiment, we performed a rescue assay by overexpressing different segments of LncPEDS1-AS in cells. Due to the length of LncPEDS1-AS, it was not feasible to construct plasmids for the overexpression of the full-length transcript. Therefore, LncPEDS1-AS was divided into three segments, which were individually overexpressed. The results indicated that overexpression of the first segment (0–2205 nt) significantly increased PEDS1 mRNA levels but did not affect the expression of PEDS1 pre-mRNA (Figs. 4G–I and S4A).

Based on these findings, we infer that LncPEDS1-AS does not function as a ‘decoy’ to protect PEDS1 mRNA or pre-mRNA from degradation. Additionally, it does not appear to influence the transcription of PEDS1 from DNA. Instead, the regulatory role of LncPEDS1-AS on PEDS1 expression primarily involves modulating the splicing of pre-mRNA into mRNA, thereby affecting the mRNA maturation process.

Subsequently, we utilised the catRAPID database to predict potential proteins that might interact with LncPEDS1-AS [22]. KEGG enrichment analysis was then performed on the genes encoding proteins with high binding probabilities [23]. The enrichment results revealed that these proteins were significantly enriched in the spliceosome pathway. This finding aligns with our previous experimental results (Fig. 4J).

To identify which protein interacting with LncPEDS1-AS, we further analysed three datasets: the top 300 genes associated with PEDS1 mRNA expression in UTUC, the top 500 proteins predicted by catRAPID to have strong binding capacities, and spliceosome-related molecules. Among these, DDX23 was identified as a common factor across all three datasets. Notably, DDX23 is a spliceosomal enzyme that is highly expressed in most cancers and is closely associated with several biological behaviours of tumour cells. On the one hand, this reflects the enhanced metabolic activity and increased mRNA synthesis characteristic of tumour cells, and on the other hand, suggests that DDX23 may influence the synthesis of multiple mRNAs (Figs. 4K–M, S4B–G and S5A–F).

In our investigation of the functional mechanisms of LncPEDS1-AS, we carried out additional work. Current research widely acknowledges that the prevalence of antisense lncRNAs varies significantly across transcriptomes; however, they constitute approximately 20% of all identified lncRNAs [17, 24]. This suggests that antisense lncRNAs not only play important roles but also exhibit heterogeneity, as observed in UTUC and UCB. To further explore this, we utilised the Lncipedia database to perform predictive and analytical evaluations of RNA-binding proteins across a broad range of lncRNA samples.

The results revealed that longer antisense lncRNAs are more likely to interact with splicing factors, which may in turn influence the expression of sense strand RNAs. This regulatory effect can be attributed to two primary factors. First, an increase in RNA length enhances the diversity and density of protein-binding sites. Second, longer lncRNAs exhibit reduced nuclear export efficiency, which facilitates their interaction with splicing-related proteins that are predominantly localised in the nucleus. Moreover, the unique complementary structure of antisense lncRNAs enables them to form complexes with one or more corresponding sense strand RNAs (Fig. S6A–C).

This characteristic endows longer antisense lncRNAs (particularly those overlapping with protein-coding genes on the antisense strand) with the ability to regulate the expression of sense strand RNAs. These findings highlight the critical role of antisense lncRNAs in gene regulation.

### Verification of LncPEDS1-AS affecting PEDS1 expression through DDX23

**Fig. 5:**
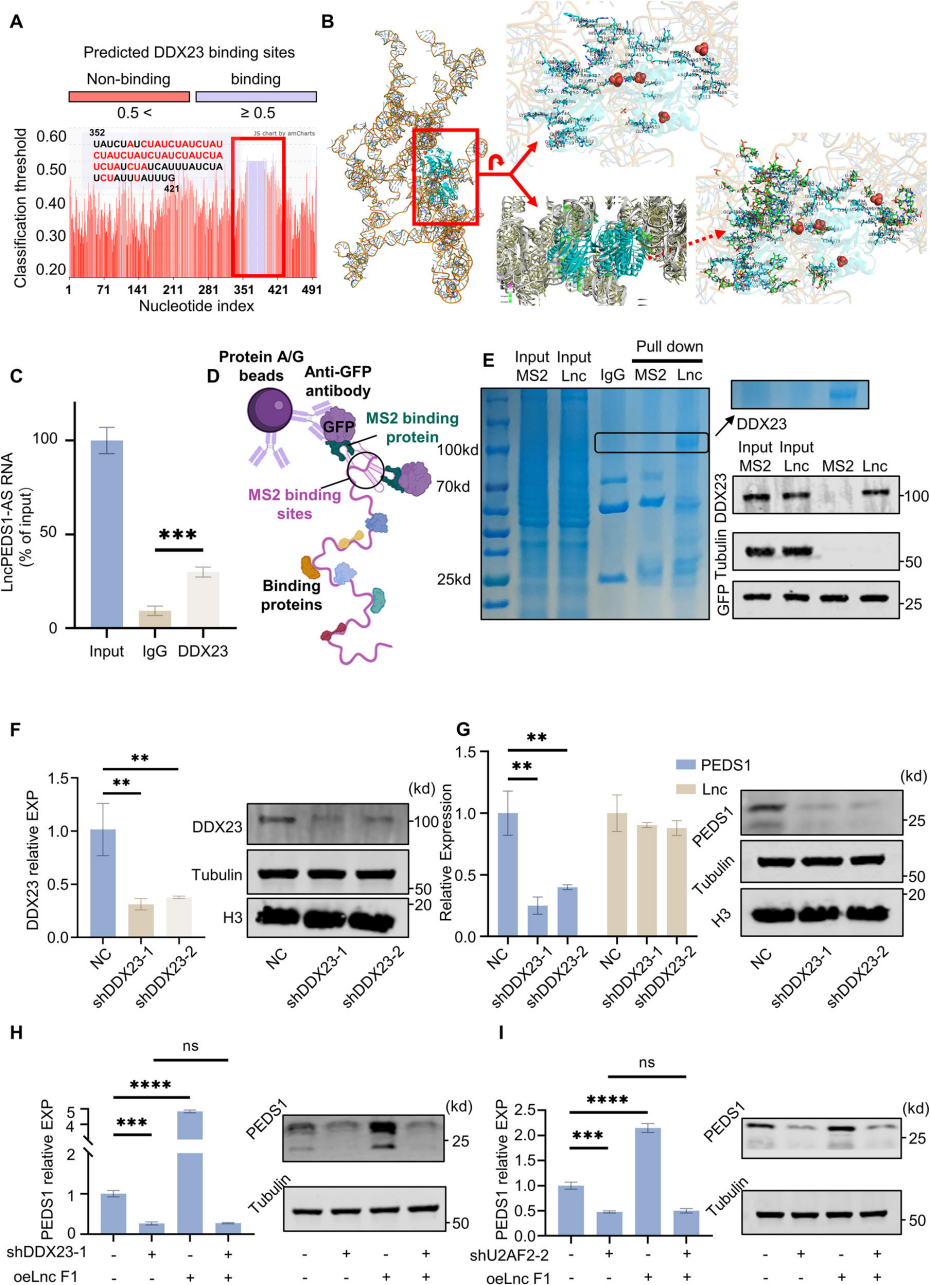
DDX23 interacts with LncPEDS1-AS to regulate PEDS1 expression. **A**, **B** Analysis of the binding potential of the primary and tertiary structures of LncPEDS1-AS with the DDX23 protein. **C** RNA immunoprecipitation analysis showing the interaction of DDX23 protein with LncPEDS1-AS. **D**, **E** MS2 RNA pull-down experiments demonstrating the presence of DDX23 among proteins bound to the first fragment of LncPEDS1-AS (Created with BioRender.com). **F** Effects of different DDX23 knockdown sites on DDX23 expression. **G** Effects of different DDX23 knockdown sites on the expression of LncPEDS1-AS and PEDS1. **H** Rescue experiments illustrating the effects of DDX23 and LncPEDS1-AS on PEDS1 expression. **I** Rescue experiments illustrating the effects of U2AF2 and LncPEDS1-AS on PEDS1 expression. (Data are shown as the mean ± SD, *n* = 3. *p* > 0.05 as ns, *p* ≤ 0.01 as **, *p* ≤ 0.001 as ***, *p* ≤ 0.0001 as ****).

To verify the existence of the LncPEDS1-AS-DDX23-PEDS1 axis, we first want to confirm whether DDX23 binds to LncPEDS1-AS. We verified the binding ability of DDX23 protein and LncPEDS1-AS in multiple protein and RNA-binding models. The results showed that whether the secondary structure sequences or the docking of tertiary structure molecules, LncPEDS1-AS shows a good binding ability with DDX23 (Figs. 5A, B and S7A, B). It is worth noting that DDX23 has the best binding ability within the first 1500nt of LncPEDS1-AS. This echoes our experimental results of overexpressing LncPEDS1-AS by sections. The RNA Immunoprecipitation results showed that there is LncPEDS1-AS in the RNA bound by DDX23, which is about 30% of the input. Considering that LncPEDS1-AS is an RNA with a length more than 6900nt, and it is easy to break, we believe this result is considerable (Fig. 5C). After that, we used the MS2-GFP-RNA pulldown to verify the proteins that bind to the first section of LncPEDS1-AS [25]. The experimental results showed that in the pull-down protein of transfected lncRNA-ms2 first section, a deep staining area appeared around 100KD. At the same time, western blot showed that the DDX23 protein existed in the pull-down protein. This shows that DDX23 can bind to LncPEDS1-AS (Fig. 5D, E).

Next, we verified whether DDX23, which binds to LncPEDS1-AS, can affect the expression of PEDS1. We knocked downDDX23 in the EJ cell line and found that the PEDS1 content in the knockdown group significantly decreased, while LncPEDS1-AS did not change significantly. This suggests that DDX23 can significantly affect the expression of PEDS1 mRNA. In addition, after the knockdown of DDX23 and the subsequent re-overexpression of the first segment of LncPEDS1-AS, we observed a marked decline in LncPEDS1-AS’s ability to promote the expression of PEDS1 mRNA (Fig. 5F–H). To further confirm that LncPEDS1-AS enhances PEDS1 mRNA expression by promoting spliceosome function through DDX23 rather than affecting PEDS1 mRNA expression through other pathways of DDX23, we also knocked down a key protein, U2AF2, in the spliceosome pathway. In the U2AF2 knockdown group, we can still see that due to the reduction of the key protein in the spliceosome, the content of PEDS1 mRNA decreased, and more importantly, the same as in the DDX23 knockdown, the ability of overexpressed lncRNA first section to promote PEDS1 mRNA content significantly weakened (Figs. 5I and S7C, D). The results of the above experiments indicate that LncPEDS1-AS promotes the splicing of PEDS1 pre-mRNA by interacting with DDX23, thereby increasing PEDS1 expression. This, in turn, enhances the resistance of UTUC tumour cells to ROS, improves their survival capacity, and contributes to the increased malignancy of UTUC.

### Exploration of the therapeutic potential of ASO drugs targeting LncPEDS1-AS

In UTUC, considering the strong association between the LncPEDS1-AS-DDX23-PEDS1 axis and prognosis, as well as our previous research findings [4], particularly the subcellular localisation of LncPEDS1-AS, we designed an ASO drug targeting this lncRNA using SmartTarget technology (RIBOBIO®). Given that the drug specifically targets LncPEDS1-AS, and the lncRNAs are generally expressed at much lower levels than mRNAs and have higher tissue specificity, LncPEDS1-AS ASO drug is expected to be more moderate, with a reduced likelihood of exacerbating systemic inflammation or inducing bone marrow suppression (Figs. S1A and S7E) [26].

**Fig. 6:**
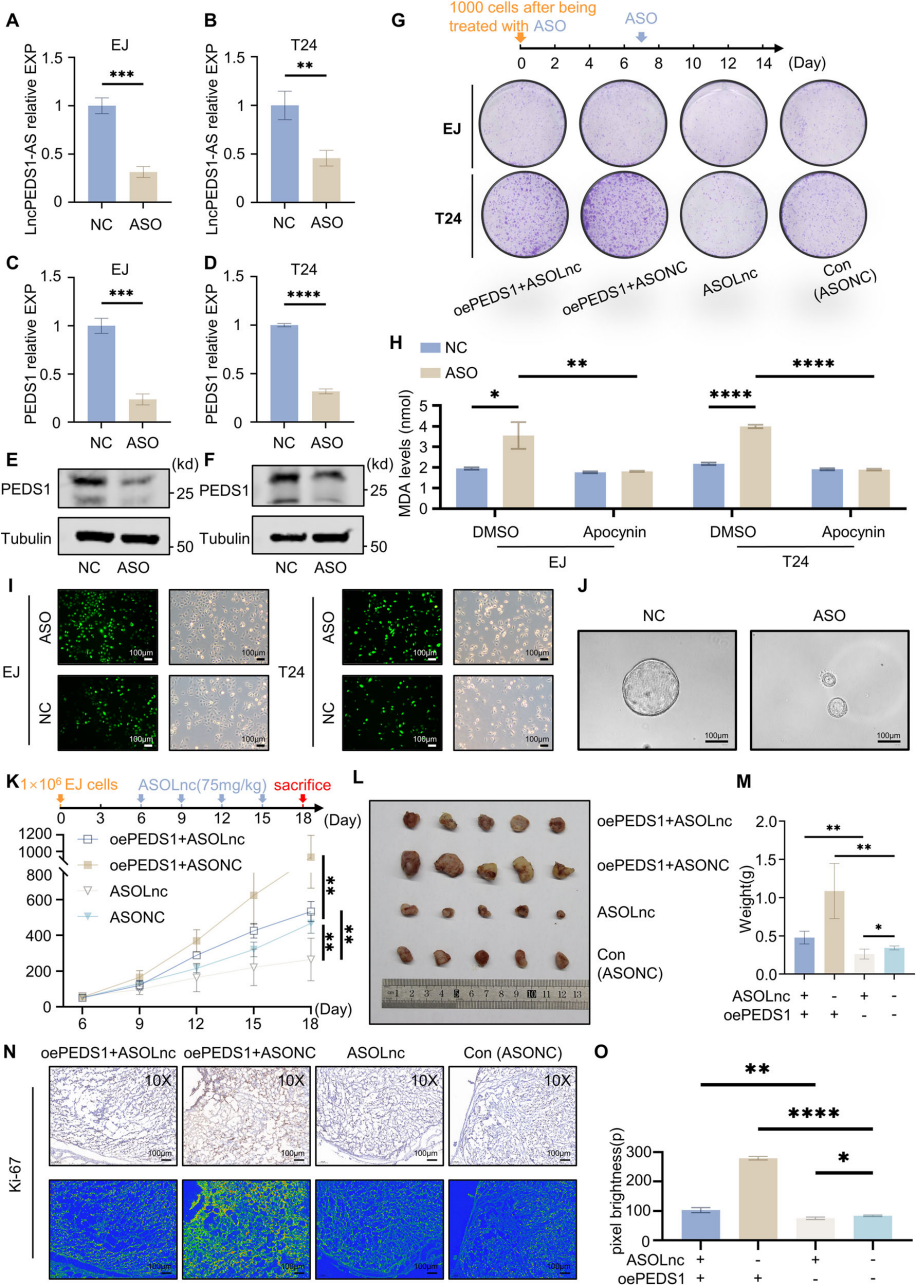
ASO drugs improve tumour prognosis by targeting LncPEDS1-AS. **A**, **B** Effects of ASO drugs on LncPEDS1-AS expression. **C**–**F** Effects of ASO drugs on PEDS1 expression. **G** Colony formation assay comparing the control and PEDS1 overexpression groups treated with ASO drugs. **H** MDA levels in control and ASO-treated cells with Fer-1 treatment. **I** Effects of ASO drugs on ROS accumulation in tumour cells. Scale bar, 100 μm. **J** Effects of ASO drugs on UTUC organoids. Scale bar, 100 μm. **K**–**M** Tumour formation experiments in mice (*n* = 5). **N**, **O** Ki67 levels in tumours from mice in different treatment groups (*n* = 5). Scale bar, 100 μm. (Data are shown as the mean ± SD, unless otherwise specified, *n* = 3. *p* ≤ 0.05 as *, *p* ≤ 0.01 as **, *p* ≤ 0.001 as ***, *p* ≤ 0.0001 as ****).

In vitro validation experiments demonstrated that the ASO drug effectively penetrated the nuclear membrane and significantly reduced the expression of LncPEDS1-AS, subsequently leading to a decrease in PEDS1 expression (Figs. 6A–F and S8A–H). Meanwhile, the ASO had no impact on the expression levels of DDX23 (Figs. S8E, F and S9A, B). Furthermore, following drug treatment, tumour cell viability was reduced, and sensitivity to ROS stress was increased (Fig. 6G–I). Organoid experiments of UTUC and primary UTUC cells experiment further demonstrated that the ASO drug effectively inhibited the growth of UTUC organoids and affect antioxidant pathways (Figs. 6J and S8G, H).

Furthermore, we assessed the drug’s therapeutic potential and safety of the drug in a nude mouse tumour xenograft model. The results indicated that this ASO drug significantly inhibited tumour growth, even under extreme conditions such as direct intravenous injection, highlighting its promise as a potential therapeutic agent (Figs. 6K–O and S9C–F).

## Discussion

UTUC and UCB are both classified as urothelial carcinomas. However, UTUC typically exhibits a poorer prognosis [2, 4]. Specifically, most bladder cancer patients undergoing conservative treatment experience relatively slow tumour progression, with progression-free survival lasting 5–10 years or even longer—an outcome rarely observed in UTUC [2]. This difference reinforces the view that UTUC is a distinct subtype of urothelial carcinoma and underscores the urgent need to investigate the molecular mechanisms contributing to its unfavourable prognosis. Current epidemiological research reveals that a significant proportion of UTUC cases is associated with inflammatory responses, and the accumulation of ROS represents a key mechanism by which inflammatory factors induce cell death. Nevertheless, tumour cells deploy negative feedback mechanisms to mitigate excessive ROS accumulation. The small subset of tumour cells that develop resistance to ROS ultimately exhibit a ‘Matthew effect’, leading to poor treatment responses and worse prognosis in UTUC [4].

Our study demonstrates that LncPEDS1-AS is highly expressed in UTUC and is closely linked to poor prognosis. By increasing the local concentration of the enzyme DDX23 within the spliceosome, LncPEDS1-AS promotes the expression of the sense-strand mRNA PEDS1, diminishing the sensitivity of tumour cells to ROS accumulation. This regulatory mechanism offers novel insights into potential therapeutic strategies for UTUC. At the same time, the unique interplay between UTUC and resistance to lipid peroxidation remains insufficiently explored, and most non-surgical treatment approaches are adapted directly from those designed for UCB. Given the tissue-specific nature of most lncRNAs, we designed ASO drugs targeting LncPEDS1-AS to enhance UTUC’s sensitivity to ROS and thereby improve its generally poor outcome (Fig. Abstract).

In addition, our investigation sheds light on the molecular mechanisms of ‘ultra-long’ antisense lncRNAs, emphasising their critical yet frequently overlooked role in regulating mRNA maturation. Most lncRNAs studied to date rarely exceed 4000 nucleotides in length; however, in our research on LncPEDS1-AS, we observed that this class of lncRNAs frequently exhibits a pronounced tendency to bind splicing factors. By analysing different lncRNAs in the Lncipedia database, we further confirm that longer antisense lncRNAs possess an enhanced capacity for binding splicing factors. This phenomenon arises primarily from their increased length, which provides more binding motifs, thereby enriching the diversity and number of RNA-binding proteins. Moreover, extended nuclear retention fosters more robust interactions with nuclear proteins, many of which are splicing factors. Notably, multicolour structural microscopy studies revealed that the well-known lncRNA MALAT1, which exceeds 10,000 nucleotides, associates with numerous splicing factors localised around nuclear speckles—empirical evidence that supports our hypothesis [27]. Furthermore, the unique transcriptional orientation and genomic location of antisense RNAs allow them to form complementary complexes with their corresponding sense-strand mRNAs, modulating maturation of those transcripts. Although our experimental work focused on LncPEDS1-AS specifically, this distinctive mechanisms among ‘ultra-long’ lncRNAs appear to be widespread yet underappreciated, calling for further research.

Our research has certain limitations that deserve consideration, we outline here to facilitate further exploration by other researchers. Firstly, although LncPEDS1-AS undoubtedly plays a vital role in the poor prognosis of UTUC, it is not the only one. Secondly, while investigating the propensity of LncPEDS1-AS to bind proteins, we noted that many spliceosomal proteins lack the corresponding immunoprecipitation-grade antibodies, forcing a reliance on computational simulations to predict binding ability. Although the interaction between the LncPEDS1-AS and DDX23 is remarkable, other binding protein certainly exist. In addition, although we performed some validation experiments using primary UTUC cells, the lack of established UTUC cell lines—combined with the fact that primary cells are prone to fibrosis and exhibit slow growth—posed significant limitations. Therefore, we conducted most of our cellular and molecular biology experiments using bladder urothelial carcinoma cell lines, which share the closest origin to UTUC.

Our findings offer important insights into the role of LncPEDS1-AS in UTUC progression and highlight its potential as a therapeutic target. Moreover, the unique characteristics of ‘ultra-long’ antisense lncRNAs identified in this study underscore their broader biological relevance, meriting further exploration in UTUC and other malignancies.

## Methods

### Bioinformatic analysis

We leveraged the ‘limma’ or ‘DEseq2’ R packages to identify differentially expressed transcripts between tumour and adjacent tissues in 69 validation samples (Data 2 and 3). The ‘clusterProfiler’ R package for KEGG pathway enrichment analysis. The ‘survminer’ R package evaluated prognostic differences based on target expression levels. The ‘survival’ and ‘timeROC’ R packages assessed the association between specific targets and survival time, testing the predictive ability of the PEDS1 expression level for UTUC prognosis at 1, 3, and 5 years. Lastly, we utilised ‘pheatmap’ and ‘ggplot2’ R packages for visualisation.

### RNA isolation and quantitative PCR (RT-qPCR)

We used TRIzol® reagent (Invitrogen) to extract Total RNA. We prepared cDNA using NovoScript® Plus All-in-one 1st Strand cDNA Synthesis SuperMix (gDNA Purge) (Novoprotein, Shanghai, China). We quantified gene transcription using the QuantStudio Three Real-Time PCR System (Thermo Fisher) and NovoStart® SYBR qPCR SuperMix Plus (Novoprotein), with GAPDH as an internal control (The primers used for RT-qPCR, along with their corresponding sequences, can be found in Table S1).

### Western blot (WB)

Total protein from tissues and cells was extracted using Solarbio’s RIPA buffer with 1% PMSF and quantified using Solarbio’ BCA kit (Solarbio, Beijing, China). The SDS-PAGE gel was prepared using Sparkjade’s kit and used for total protein separation (Sparkjade, Shandong Sparkjade Biotechnology Co.). Proteins were transferred to a Millipore nitrocellulose membrane, blocked, and incubated overnight with the primary antibody. After washing, the membrane was incubated with either goat anti-rabbit IgG or goat anti-mouse IgG. The Odyssey CLx infrared imaging system from Gene Company was used for target protein band detection. (Primary antibodies: anti-PEDS1 ActivAb Cat# K005799P, anti-PMP70 Abcam Cat# ab85550, anti-DDX23 Aviva Systems Biology Cat# OAGA04957, anti-GFP Abbkine Cat# ABT2020, anti-H3 Abbkine Cat# ABL1070, anti-U2AF2 SantaCruz Cat# sc-53942, anti-NRF2 Proteintech Cat# 16396-1-AP, anti-ACSL4 Epizyme Cat# 99M02K23, anti-FSP1 Proteintech Cat# 20886-1-AP, anti-GPX4 Proteintech Cat# 30388-1-AP, anti-SOD1 Epizyme Cat# 33L42M95, anti-GAPDH Proteintech Cat# 60004-1-Ig, anti-Tubulin CST Cat# 2128. Further details of the antibodies used are listed in the Table S2).

### Cell culture and transfection

All cell cultures were consistently kept at a temperature of 37 °C and a CO2 concentration of 5%, with each medium further supplemented with 10% foetal bovine serum (Gibco, Waltham, MA, USA).

Cells were transfected with plasmid DNA using Lipofectamine 3000 (Thermo Fisher), and siRNA was introduced using the jetPRIME® Versatile DNA transfection reagent (Polyplus Transfection), following the manufacturer’s instructions. Viral packaging was commenced in the HEK-293T cells after co-transfection of the plasmid with the packaging plasmid psPAX2 and envelope plasmid.

According to the manufacturer’s instructions, the ASO was used at a concentration of 50 nM in cell-based experiments and was delivered in combination with riboFECT™ CP Reagent, as recommended, to enhance its cellular uptake.

pMD2.G, facilitated by Lipofectamine 3000. Viruses were gathered approximately 2 days after the transfection. The target cells were subsequently exposed to the recombinant lentivirus-transducing units, alongside 8 μg/ml Polybrene (Beyotime). (The shRNA sequences and cell lines are shown in Table S3).

### ROS fluorescence probe assay

The ROS Assay Kit (DCFH-DA) (MKBio, Shanghai, China) was used to complement and validate the content of intracellular ROS. A total of 2.5 × 10^5^ cells to be tested in each well were placed in a six-well plate, and the culture medium was extracted before adding the 10 mM of working solution with phosphate-buffered saline (PBS). They were incubated at room temperature for 40 min. The staining working solution was removed, the cells were washed once with preheated PBS, and then washed once with preheated cell culture medium. The preheated cell culture medium was added again before evaluation using a microscope (Leica DM IRB, Wetzlar, Germany).

### Wound-healing assay

5 × 10^5^ urothelial carcinoma cells were placed and cultured in RPMI-1640 with FBS. When the cell density was 80-100%, a wound was made in the cell monolayer, and the prime image was immediately photographed. Then, cells placed in six-well plates were cultured for another 24 or 48 h, and the corresponding images were photographed immediately. The area of cell migration was our primary outcome measure used to analyse the migration ability of cell lines.

### Transwell assay

Cell invasion ability was assessed using the 8 μm pore size Transwell assay (Corning Inc., Corning, NY, USA). After cells were digested with trypsin, they were collected and placed in the upper chamber containing 1 × 10^5^ cells where matrix gel was added, and then the medium in each well was supplemented to 200 μL. The upper chamber was placed in a 24-well plate with RPMI-1640 medium containing 20% FBS. After incubation at 37 °C and 5% CO_2_ for 24 h, the upper chamber was stained and analysed.

### Colony formation assay

Approximately 3000 cells were cultured with 2 ml 1640 media containing 10% FBS in each well of a 6-well plate. The cells were cultured for 5 days and fixed with paraformaldehyde for 30 min. The cell colonies were then stained with crystal violet for 30 min and subsequently washed with distilled water.

### ROS sensitivity assay

Cells were seeded into each well of a 6-well plate at a density of 1 × 10^5^ cells per well, according to the experimental groups. After 6 h, once the cells were fully adhered, the culture medium was replaced, and RSL3 (Targetmol Cat# T3646) was added at a final concentration of 5 µM. Following a 12-h incubation, the cells were treated with paraformaldehyde for 30 min. And then stained with crystal violet staining solution for 30 min and subsequently washed with distilled water.

### Cell counting kit-8 assay

The cell counting kit-8 (CCK8) assay (MCE, NJ, USA) was used to assess the proliferative viability of tumour cell lines. 3000 cells were cultured in 96-well plates filled with 100 μL RPMI-1640 and 10% FBS for the corresponding time. Then, 10 µL of the CCK8 reagent was supplemented to each well 1 h after cell placement. The absorbance in each well was measured with a DNM-9606 microplate reader (Perlong, Beijing, China) at 450 nm every 24 h.

### Immunohistochemistry

Paraffin-embedded tissues were sliced into 5-μm sections and observed for target proteins expression after immunohistochemistry staining. These sections were dyed with diluted anti- the target protein antibody (1:500) and incubated at 4 °C overnight; then, they were washed three times with fresh PBS solutions and immediately incubated with biotin-labelled second antibody for immunohistochemistry (GB23303; Servicebio, Wuhan, China) for 50 min at room temperature. The positive cells were monitored using diaminobenzidine solution (K5007; DAKO, Santa Clara, CA, USA) by direct viewing. The processed slides were observed using a light microscope (Leica DM2700).

### MDA assay

The micro-MDA assay kit (Solarbio) was used for lipid oxidation measurement in cell membranes. Cells were collected, centrifuged, and treated with Reagent I, then sonicated and re-centrifuged. The supernatant was collected and chilled. The spectrophotometer and microplate reader, set to 415 nm, was preheated and zeroed with distilled water. IFU-directed additions of samples and reagents I, II, and III were made. The mixture was heated at 100 °C for 60 min, cooled on ice, and re-centrifuged at 10,000 × *g*.

### Immunofluorescence staining

Cell slides were treated with xylene for 10 min, then sequentially dehydrated in ethanol solutions of decreasing concentration and washed with distilled water. Slides were then incubated with 0.5% Triton X-100 buffer, washed with PBS, and sealed with goat serum. After overnight incubation with diluted primary antibodies at 4 °C, slides were washed and incubated with the corresponding secondary antibody. Following DAPI treatment, the slides were washed, fixed with anti-fluorescence agent, and examined under a fluorescence microscope.

### RNA fluorescence in situ hybridisation

RNA Fluorescence in situ hybridisation was performed according to the manufacturer’s instructions. Cy3-labelled LncPEDS1-AS probes were designed and synthesised by RiboBio (Guangzhou, China). The signals of LncPEDS1-AS were detected by Fluorescent in Situ Hybridization Kit (RiboBio, Guangzhou, China). The images were captured using Nikon A1Si Laser Scanning Confocal Microscope (Nikon Instruments Inc., Japan).

### Molecular docking

The Bclab-pridictor and catRAPID databases were used for protein RNA-binding prediction and secondary structure prediction, respectively [22, 28]. RNA tertiary structure docking was verified using the HDOCK server and 3dRNA server for molecular docking and RNA modelling. Analysis of protein-protein docking, binding activities, and proximate amino acid residue interactions were conducted with HDOCK. The DDX23 protein crystal structure was sourced from the PDB RCSB. PyMOL and Discovery Studio software were employed for ligand and protein isolation, dehydration, organic material removal, and analysis of hydrogen bonds and hydrophobic forces. PyMOL also illustrated interactions between LncPEDS1-AS and DDX23 amino acid residues.

### RNA immunoprecipitation (RIP)

Cells seeded in a 15-cm dish at 70–80% confluency were cross-linked by ultraviolet light at 254 nm (200 J/cm^2^), then harvested and lysated. RNA immunoprecipitation (RIP) assay was performed according to the instructions of the Magna RIP RNA Binding Protein Immunoprecipitation Kit (Millipore, USA), using an antibody specific for DDX23, or a corresponding rabbit IgG (NI01, Millipore). Input and co-immunoprecipitated RNAs were extracted and used NovoScript® Plus All-in-one 1st Strand cDNA Synthesis SuperMix for qRT-PCR.

### MS2 RNA pulldown

MS2-RIP assays were performed according to the instructions of a Millipore Magna RIP Kit (Millipore, Darmstadt, Germany). The 5 × MS2 stem loop was fused to the LncPEDS1-AS-F1 plasmid. The indicated cells were co-transfected with the target RNA expression vector or 5× MS2 stem loop fused with the MS2-GFP vector, and these cells were lysed with RIP lysis buffer containing protease and RNase inhibitor. After the cell lysate was cleared by centrifugation, the supernatant was inoculated with anti-GFP/IgG antibody-conjugated beads overnight at 4 °C. Then, the binding complexes were thoroughly washed, eluted, purified and analysed by western blot. (The MS2 loop is shown in Table S4).

### Human samples

Primary UTUC malignant tissue samples and their corresponding adjacent normal tissues were collected with approval from the Research Ethics Board (2023-KY-0319), and all patients provided written informed consent. The samples were sourced from the First Affiliated Hospital of Zhengzhou University, Henan, China (The Urological Diseases Sub-bank of the Tissue Sample Bank at the First Affiliated Hospital of Zhengzhou University) (Data 2). mRNA expression was measured using RT-qPCR, while protein levels were determined via Western blot analysis.

### Organoid culture

Tumour specimens (1–3 mm^3^) were obtained intraoperatively from patients diagnosed with UTUC associated with aristolochic acid exposure (2023-KY-0319). The tissue was incubated at 37 °C for 1 h in Advanced DMEM/F12 (AdDMEM/F12) supplemented with collagenase II and the ROCK inhibitor Y-27632. After enzymatic digestion, the tissue suspension was centrifuged at 200 × *g* for 5 min, and the pellet was washed once with AdDMEM/F12 to remove residual enzymes and debris. The washed tissue was then resuspended in 5 mL of TrypLE Express containing Y-27632 and incubated at 37 °C for 5 min to achieve further dissociation into single cells. The enzymatic reaction was neutralised by adding 10 mL of AdDMEM/F12 supplemented with 20% FBS and 1% penicillin–streptomycin. The resulting cell suspension was passed through a 70 μm cell strainer to remove undigested debris. After another round of centrifugation (200 × *g*, 5 min), the cell pellet was resuspended in 200 μL of cold Matrigel. Approximately 30 μL of the Matrigel–cell mixture was plated into each well of a pre-warmed 24-well plate. Once the Matrigel had solidified at 37 °C (10–15 min), 2.5 mL of complete human UTUC organoid culture medium was added to each well (UTUC organoid media recipe is shown in Table S5) [29]. Organoids were maintained in a humidified incubator at 37 °C with 5% CO_2_, and the culture medium was refreshed every 2–3 days.

### In vivo tumour xenograft model

In an animal study, 23 female nude mice (20 used for the experiment and 3 reserved as backups, which were ultimately not utilised) aged 4–5 weeks (Sibeifu Company, Beijing, China) were included. Twenty mice were randomly assigned to receive subcutaneous injections of Matrigel suspensions containing EJ cells (either blank control or PEDS1 overexpression, 2 × 10^6^ cells), with 10 mice per group. One week after injection, half of the mice in each group (5:5/5:5) received peritumoral injections (peritumoral injection) of an ASO drug (In accordance with the manufacturer’s instructions, the injection concentration of the ASO was 75 mg/kg), while the remaining mice were treated with a control ASO. The injections were administered every 3 days starting from the second week. On day 18, all mice were sacrificed, and the tumours were excised and photographed. Researchers were blinded to the measured samples group when assessing the tumour size of the mice.

In addition, blood and organ samples were collected from mice in the ASONC and ASOLnc groups within the xenograft model to evaluate the safety profile of the ASO therapy. To further assess the potential risks associated with direct intravenous administration of ASOs, we also included two additional groups: one group of mice (*n* = 5) received intravenous injections of normal saline, while another group (*n* = 5) received direct intravenous injections of the ASO compound. Blood and organ samples from both groups were collected for comparative safety analysis.

The experiment complied with Chinese National Institutes of Health guidelines and was approved by the Ethics Committee for Animal Experiments of the First Affiliated Hospital of Zhengzhou University (2023-KY-0319).

### Statistical analysis

Statistical analyses were performed in GraphPad Prism v9.0 and R v4.0.3. Continuous variables are presented as mean ± standard deviation, and their distributional assumptions were assessed with the Shapiro–Wilk test for normality and the Brown–Forsythe test for homogeneity of variances; if either assumption failed, the relevant non-parametric alternative was selected. Normally distributed data arising from two independent samples were compared with a two-tailed, unpaired Student’s *t*-test, whereas paired observations (e.g. before-and-after measurements or matched subjects) were analysed using a two-tailed, paired Student’s *t*-test. Effect sizes for independent-sample *t*-tests are reported as Cohen’s *d* with 95% confidence intervals, and for paired designs as Cohen’s *d*z. When normality was violated, independent and paired comparisons were undertaken with the Mann–Whitney *U* test and the Wilcoxon signed-rank test, respectively. Each experiment comprised three independent biological replicates (*n* = 3) unless stated otherwise, and no data were excluded. All p-values are two-sided; statistical significance was accepted at *p* < 0.05, with the Benjamini–Hochberg procedure employed to control the false-discovery rate when multiple related hypotheses were tested.

## Supplementary information

Supplementary Figure

Supplementary Table

Dataset 1

Dataset 2

Dataset 3

Original Western Blots

## Data Availability

All the data supporting this study’s findings are available from the corresponding authors upon reasonable request. The matrix of bulk RNA-seq is deposited in the supplementary file (Data 3).

## References

[1] Gokmen MR, Lord GM. Aristolochic acid nephropathy. BMJ. 2012;344:e4000. 22705815 10.1136/bmj.e4000

[2] Roupret M, Seisen T, Birtle AJ, Capoun O, Comperat EM, Dominguez-Escrig JL, et al. European association of urology guidelines on upper urinary tract urothelial carcinoma: 2023 update. Eur Urol. 2023;84:49–64. 36967359 10.1016/j.eururo.2023.03.013

[3] Yang HY, Chen PC, Wang JD. Chinese herbs containing aristolochic acid associated with renal failure and urothelial carcinoma: a review from epidemiologic observations to causal inference. Biomed Res Int. 2014;2014:569325. 25431765 10.1155/2014/569325 PMC4241283

[4] Li K, Huang Z, Xie G, Huang B, Song L, Zhang Y, et al. Transcriptomic insights into UTUC: role of inflammatory fibrosis and potential for personalized treatment. J Transl Med. 2024;22:24. 38183115 10.1186/s12967-023-04815-y PMC10768331

[5] Wang Z, Shi H, Xu Y, Fang Y, Song J, Jiang W, et al. Intravesical therapy for upper urinary tract urothelial carcinoma: a comprehensive review. Cancers. 2023;15:5020. 37894387 10.3390/cancers15205020 PMC10605447

[6] Anger EE, Yu F, Li J. Aristolochic acid-induced nephrotoxicity: molecular mechanisms and potential protective approaches. Int J Mol Sci. 2020;21:1157. 32050524 10.3390/ijms21031157 PMC7043226

[7] Liu Y, Liu Y, Ye S, Feng H, Ma L. A new ferroptosis-related signature model including messenger RNAs and long non-coding RNAs predicts the prognosis of gastric cancer patients. J Transl Int Med. 2023;11:145–55. 38025952 10.2478/jtim-2023-0089 PMC10680379

[8] Yuan Y, Han X, Zhao X, Zhang H, Vinograd A, Bi X, et al. Circulating exosome long non-coding RNAs are associated with atrial structural remodeling by increasing systemic inflammation in atrial fibrillation patients. J Transl Int Med. 2024;12:106–18. 38525437 10.2478/jtim-2023-0129 PMC10956728

[9] Su T, Zhang N, Wang T, Zeng J, Li W, Han L, et al. Super enhancer-regulated LncRNA LINC01089 induces alternative splicing of DIAPH3 to drive hepatocellular carcinoma metastasis. Cancer Res. 2023;83:4080–94. 37756562 10.1158/0008-5472.CAN-23-0544

[10] Zhu Y, Huang C, Zhang C, Zhou Y, Zhao E, Zhang Y, et al. LncRNA MIR200CHG inhibits EMT in gastric cancer by stabilizing miR-200c from target-directed miRNA degradation. Nat Commun. 2023;14:8141. 38065939 10.1038/s41467-023-43974-w PMC10709323

[11] Bridges MC, Daulagala AC, Kourtidis A. LNCcation: lncRNA localization and function. J Cell Biol. 2021;220:e202009045. 33464299 10.1083/jcb.202009045 PMC7816648

[12] Herman AB, Tsitsipatis D, Gorospe M. Integrated lncRNA function upon genomic and epigenomic regulation. Mol Cell. 2022;82:2252–66. 35714586 10.1016/j.molcel.2022.05.027 PMC9219586

[13] Bian Z, Yang F, Xu P, Gao G, Yang C, Cao Y, et al. LINC01852 inhibits the tumorigenesis and chemoresistance in colorectal cancer by suppressing SRSF5-mediated alternative splicing of PKM. Mol Cancer. 2024;23:23. 38263157 10.1186/s12943-024-01939-7 PMC10807094

[14] Kansara S, Sawant P, Kaur T, Garg M, Pandey AK. LncRNA-mediated orchestrations of alternative splicing in the landscape of breast cancer. Biochim Biophys Acta Gene Regul Mech. 2024;1867:195017. 38341138 10.1016/j.bbagrm.2024.195017

[15] Zhan X, Yan C, Zhang X, Lei J, Shi Y. Structures of the human pre-catalytic spliceosome and its precursor spliceosome. Cell Res. 2018;28:1129–40. 30315277 10.1038/s41422-018-0094-7 PMC6274647

[16] Wilkinson ME, Charenton C, Nagai K. RNA splicing by the spliceosome. Annu Rev Biochem. 2020;89:359–88. 31794245 10.1146/annurev-biochem-091719-064225

[17] Liu B, Xiang W, Liu J, Tang J, Wang J, Liu B, et al. The regulatory role of antisense lncRNAs in cancer. Cancer Cell Int. 2021;21:459. 34461912 10.1186/s12935-021-02168-4 PMC8404292

[18] Barretina J, Caponigro G, Stransky N, Venkatesan K, Margolin AA, Kim S, et al. The Cancer Cell Line Encyclopedia enables predictive modelling of anticancer drug sensitivity. Nature. 2012;483:603–7. 22460905 10.1038/nature11003 PMC3320027

[19] Zou Y, Henry WS, Ricq EL, Graham ET, Phadnis VV, Maretich P, et al. Plasticity of ether lipids promotes ferroptosis susceptibility and evasion. Nature. 2020;585:603–8. 32939090 10.1038/s41586-020-2732-8 PMC8051864

[20] Cui W, Liu D, Gu W, Chu B. Peroxisome-driven ether-linked phospholipids biosynthesis is essential for ferroptosis. Cell Death Differ. 2021;28:2536–51. 33731874 10.1038/s41418-021-00769-0 PMC8329287

[21] Wainberg M, Kamber RA, Balsubramani A, Meyers RM, Sinnott-Armstrong N, Hornburg D, et al. A genome-wide atlas of co-essential modules assigns function to uncharacterized genes. Nat Genet. 2021;53:638–49. 33859415 10.1038/s41588-021-00840-z PMC8763319

[22] Armaos A, Colantoni A, Proietti G, Rupert J, Tartaglia GG. catRAPID omics v2.0: going deeper and wider in the prediction of protein-RNA interactions. Nucleic Acids Res. 2021;49:W72–9. 34086933 10.1093/nar/gkab393 PMC8262727

[23] Kanehisa M, Goto S. KEGG: Kyoto Encyclopedia of Genes and Genomes. Nucleic Acids Res. 2000;28:27–30. 10592173 10.1093/nar/28.1.27 PMC102409

[24] Volders PJ, Anckaert J, Verheggen K, Nuytens J, Martens L, Mestdagh P, et al. LNCipedia 5: towards a reference set of human long non-coding RNAs. Nucleic Acids Res. 2019;47:D135–9. 30371849 10.1093/nar/gky1031 PMC6323963

[25] Yang L, Yin H, Chen Y, Pan C, Hang H, Lu Y, et al. Low expression of PEBP1P2 promotes metastasis of clear cell renal cell carcinoma by post-transcriptional regulation of PEBP1 and KLF13 mRNA. Exp Hematol Oncol. 2022;11:87. 36348434 10.1186/s40164-022-00346-2 PMC9644627

[26] Arroyo AB, Tyrkalska SD, Bastida-Martinez E, Monera-Girona AJ, Canton-Sandoval J, Bernal-Carrion M, et al. Peds1 deficiency in zebrafish results in myeloid cell apoptosis and exacerbated inflammation. Cell Death Discov. 2024;10:388. 39209813 10.1038/s41420-024-02141-w PMC11362147

[27] West JA, Davis CP, Sunwoo H, Simon MD, Sadreyev RI, Wang PI, et al. The long noncoding RNAs NEAT1 and MALAT1 bind active chromatin sites. Mol Cell. 2014;55:791–802. 25155612 10.1016/j.molcel.2014.07.012 PMC4428586

[28] Tuvshinjargal N, Lee W, Park B, Han K. PRIdictor: protein-RNA interaction predictor. Biosystems. 2016;139:17–22. 26607710 10.1016/j.biosystems.2015.10.004

[29] Li Z, Xu H, Gong Y, Chen W, Zhan Y, Yu L, et al. Patient-derived upper tract urothelial carcinoma organoids as a platform for drug screening. Adv Sci. 2022;9:e2103999. 10.1002/advs.202103999 PMC8811809 34914855

