# Supplementary material for: LncPEDS1-AS promotes UTUC resistance to lipid peroxidation by regulating PEDS1 expression via DDX23
Source: Cell Death Dis. 2025 Dec 8;17(1):87. doi: 10.1038/s41419-025-08293-6 (PMC12830725; doi:10.1038/s41419-025-08293-6)
Supplement: Supplementary file 1 — Supplementary Figure [file 41419_2025_8293_MOESM1_ESM.docx]

**LncPEDS1-AS Promotes UTUC Resistance to Lipid Peroxidation by Regulating PEDS1 Expression via DDX23**

**Guanru Li, Erwei Zhang, Zhiyu Wang,** **Zhijin Zhang, Yuke Zhang, Shichen Di, Jingyi Lu, Shun Cao, Guoqing Xie, Yu Zhang, Keqiang Li**

**Information**

**Supplementary Figure 1 （Supplemental to Figure. 1 and Figure. 2）**

**Supplementary Figure 2 （Supplemental to Figure.3）**

**Supplementary Figure 3 （Supplemental to Figure.3）**

**Supplementary Figure 4 （Supplemental to Figure. 4）**

**Supplementary Figure 5 （Supplemental to Figure. 4）**

**Supplementary Figure 6 （Supplemental to Figure. 4）**

**Supplementary Figure 7 （Supplemental to Figure. 5 and Figure. 6）**

**Supplementary Figure 8 （Supplemental to Figure. 6）**

**Supplementary Figure 9 （Supplemental to Figure. 6）**

**
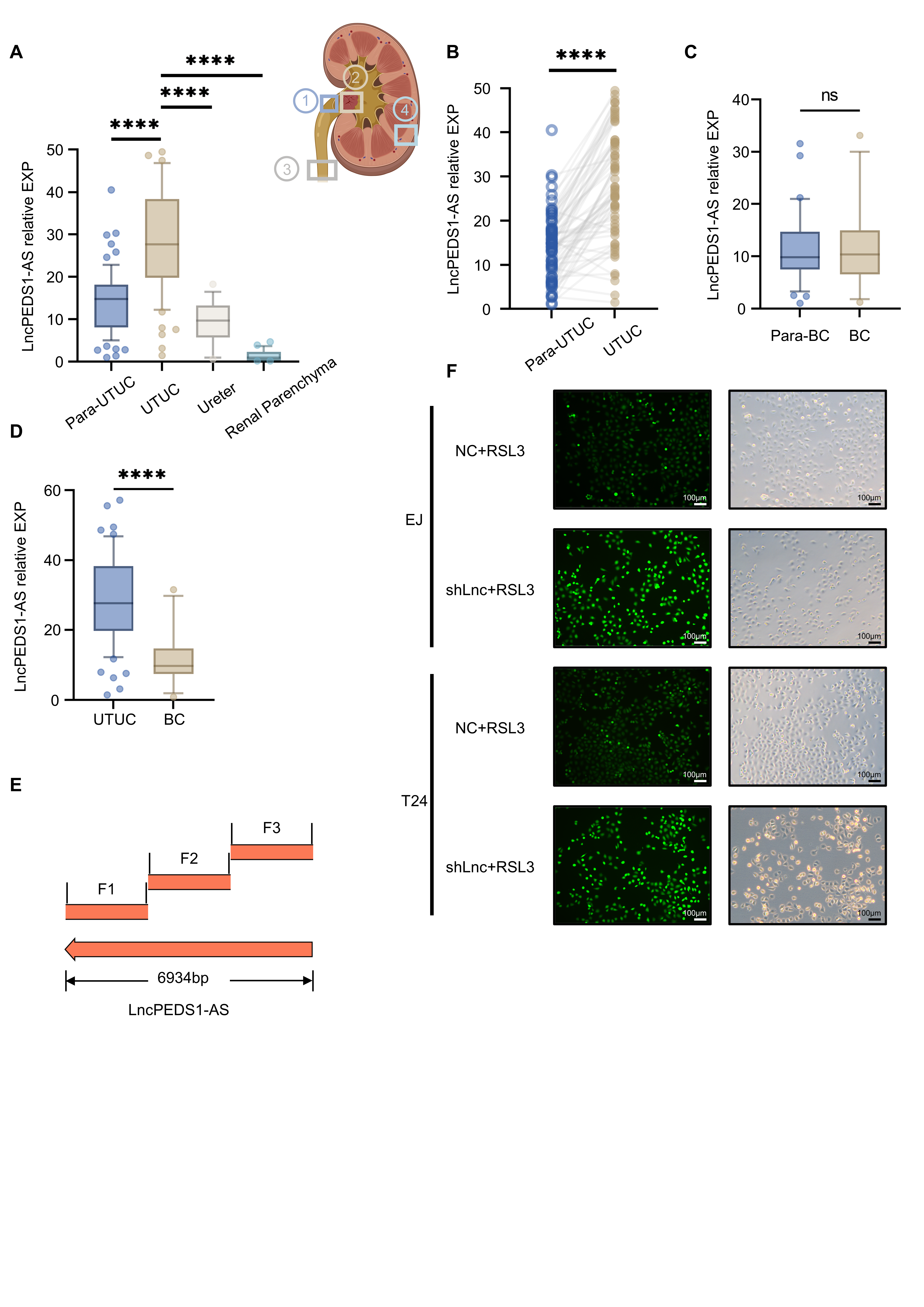
**

**Supplementary Figure 1.**

(A) Relative expression levels of LncPEDS1-AS in UTUC and in tissues from multiple adjacent urinary organs (UTUC n = 69, Ureter n = 15, Renal Parenchyma n = 25). (B) Differential expression of LncPEDS1-AS in tumour and adjacent normal tissues of UTUC patients (n = 69). (C) Expression of LncPEDS1-AS in BC and paracarcinomatous tissue (n = 34). (D) Expression of LncPEDS1-AS in UTUC and BC tissue samples (BC n = 34, UTUC n = 69). (E) Schematic representation of the LncPEDS1-AS structure. (F) Intracellular ROS levels in the LncPEDS1-AS knockdown group and the control group following RSL3 treatment. Scale bar, 100 μm. (*p* ≤ 0.0001 as ****)


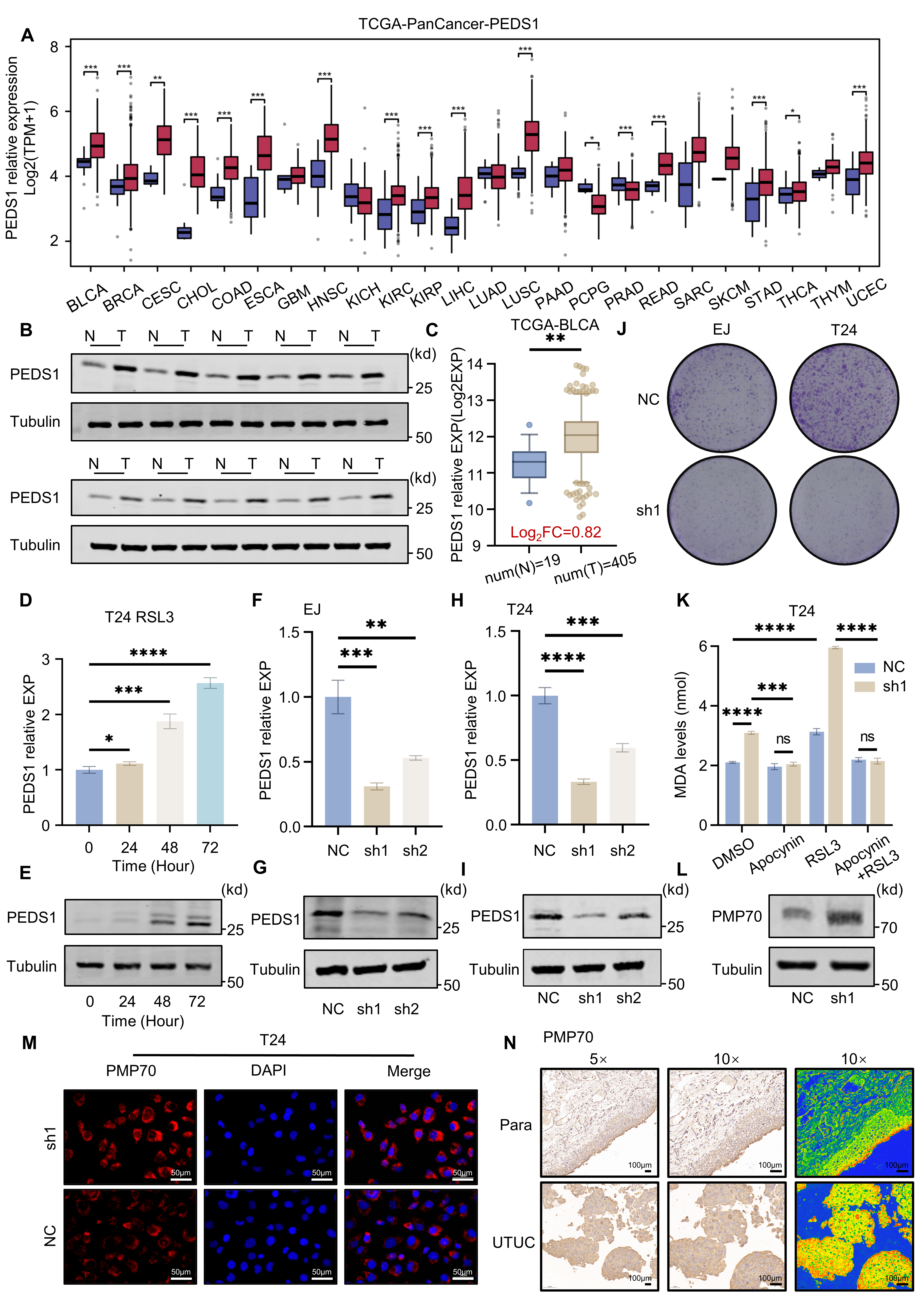


**Supplementary Figure 2.**

(A) Pan-cancer expression profile of PEDS1. (B) Levels of PEDS1 protein in UTUC samples and adjacent normal tissue samples (C) Levels of PEDS1 in TCGA bladder cancer samples and adjacent normal tissue samples (n = 424). (D–E) Effect of different RSL3 treatment durations on PEDS1 expression. (F–I) Knockdown of PEDS1 in EJ and T24 cells. (J) Colony formation assay comparing control and PEDS1 knockdown groups. (K) MDA content in control and PEDS1 knockdown cells after treatment with Apocynin and RSL3. (L–M) Expression levels of PMP70 in control and PEDS1 knockdown T24 cells. Scale bar, 50 μm. (N) Expression of PMP70 in UTUC sample and adjacent normal tissue sample. Scale bar, 100 μm. (Data are shown as the mean ± SD, unless otherwise specified, n = 3. *p* ≤ 0.05 as *, *p* ≤ 0.01 as **, *p* ≤ 0.001 as ***, *p* ≤ 0.0001 as ****)


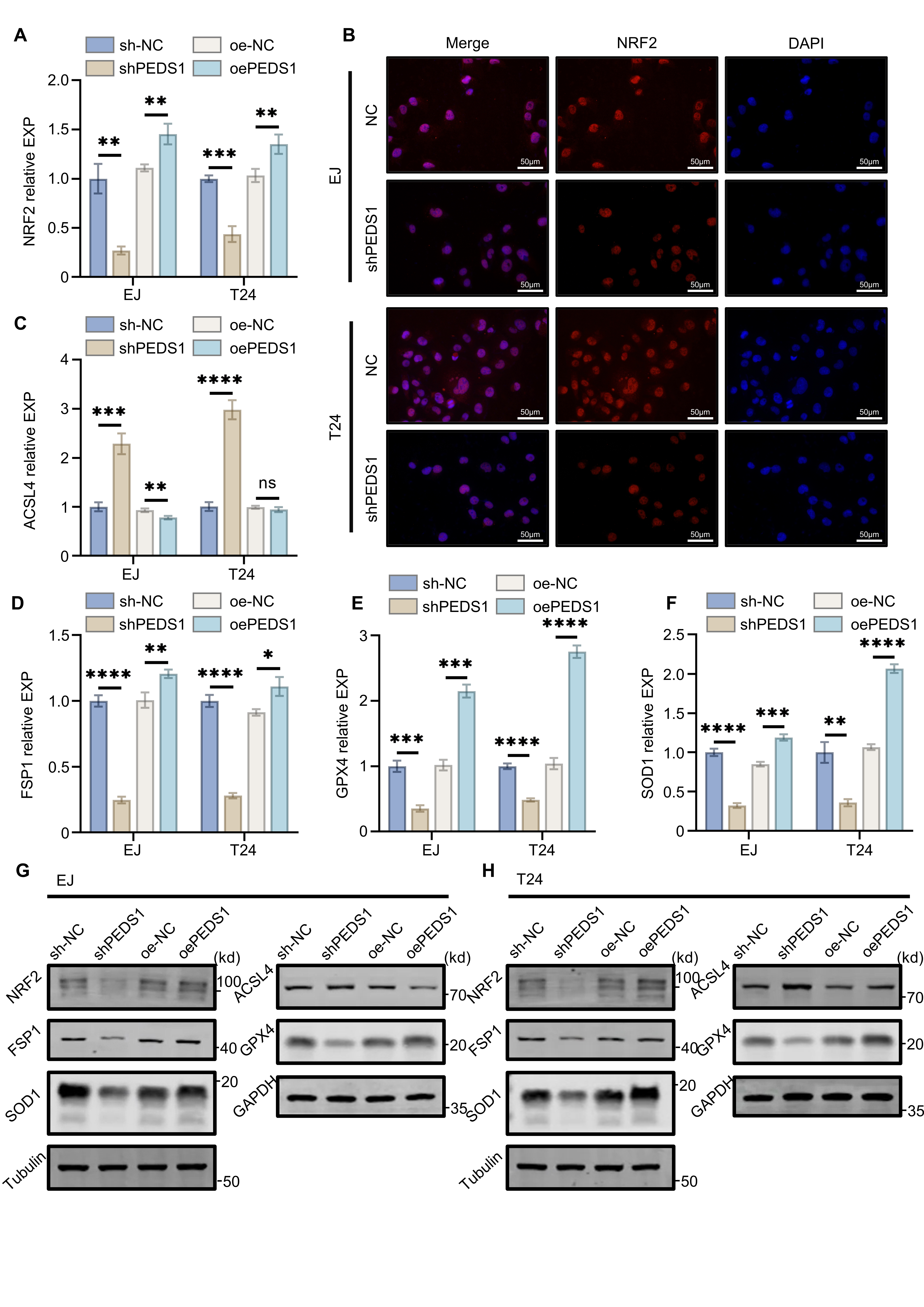
**Supplementary Figure 3.**

(A-H) Alterations in key molecules across multiple antioxidant pathways in EJ and T24 cells with PEDS1 knock-down or overexpression. Scale bar, 50 μm. (Data are shown as the mean ± SD, n = 3. *p* ≤ 0.05 as *, *p* ≤ 0.01 as **, *p* ≤ 0.001 as ***, *p* ≤ 0.0001 as ****)


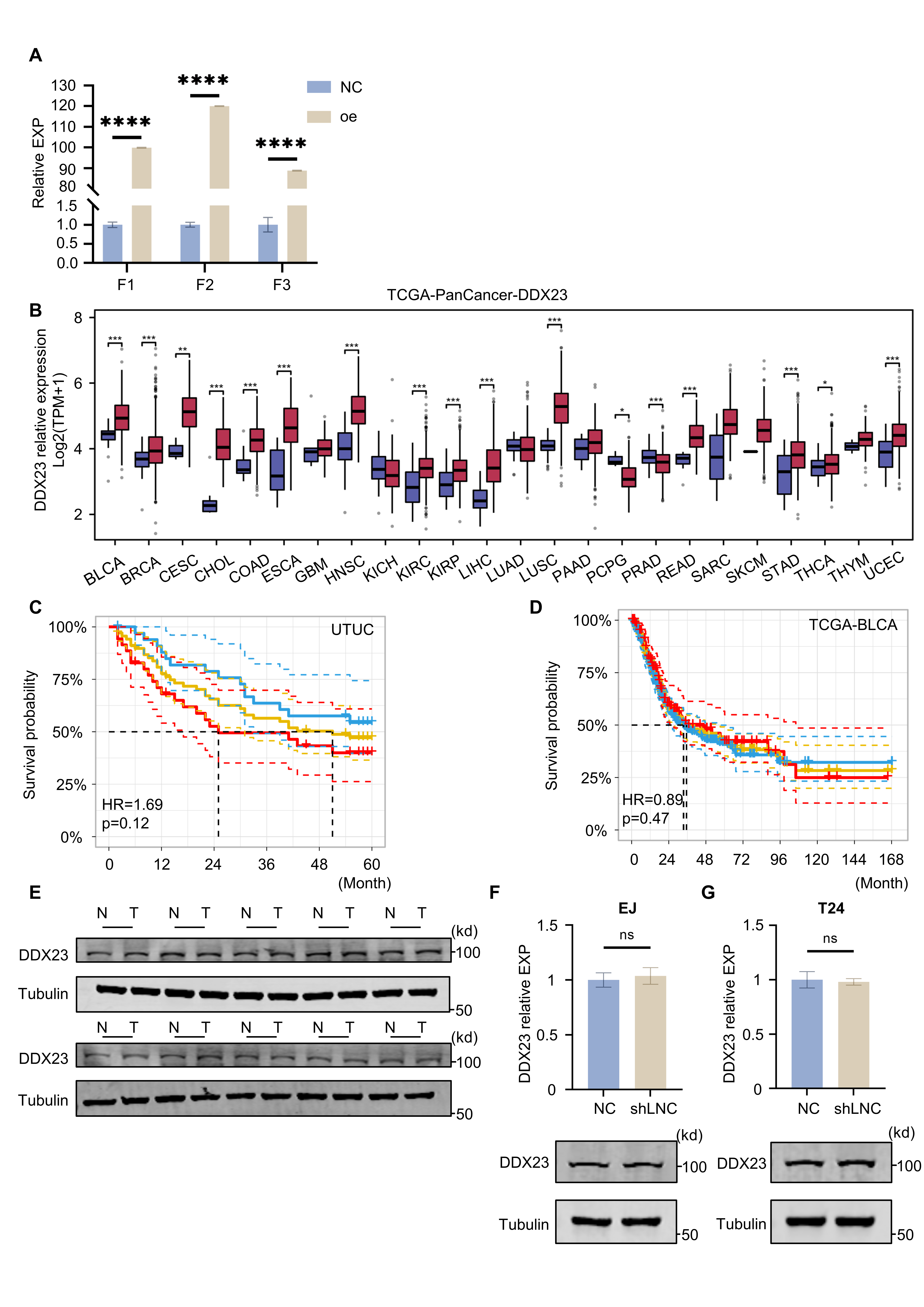


**Supplementary Figure 4.**

(A) Expression levels of F1, F2, and F3 in the overexpression groups and control groups for F1, F2, and F3, respectively. (B) Pan-cancer expression profile of DDX23. (C–D) Correlation between DDX23 expression levels and prognosis in UTUC and bladder cancer (UTUC n = 69, TCGA-BLCA n = 424). (E) Levels of DDX23 protein in UTUC samples and adjacent normal tissue samples. (F-G) Effect of LncPEDS1-AS knockdown on DDX23 expression levels. (Data are shown as the mean ± SD, unless otherwise specified, n = 3. *p* ≤ 0.05 as *, *p* ≤ 0.01 as **, *p* ≤ 0.001 as ***, *p* ≤ 0.0001 as ****)


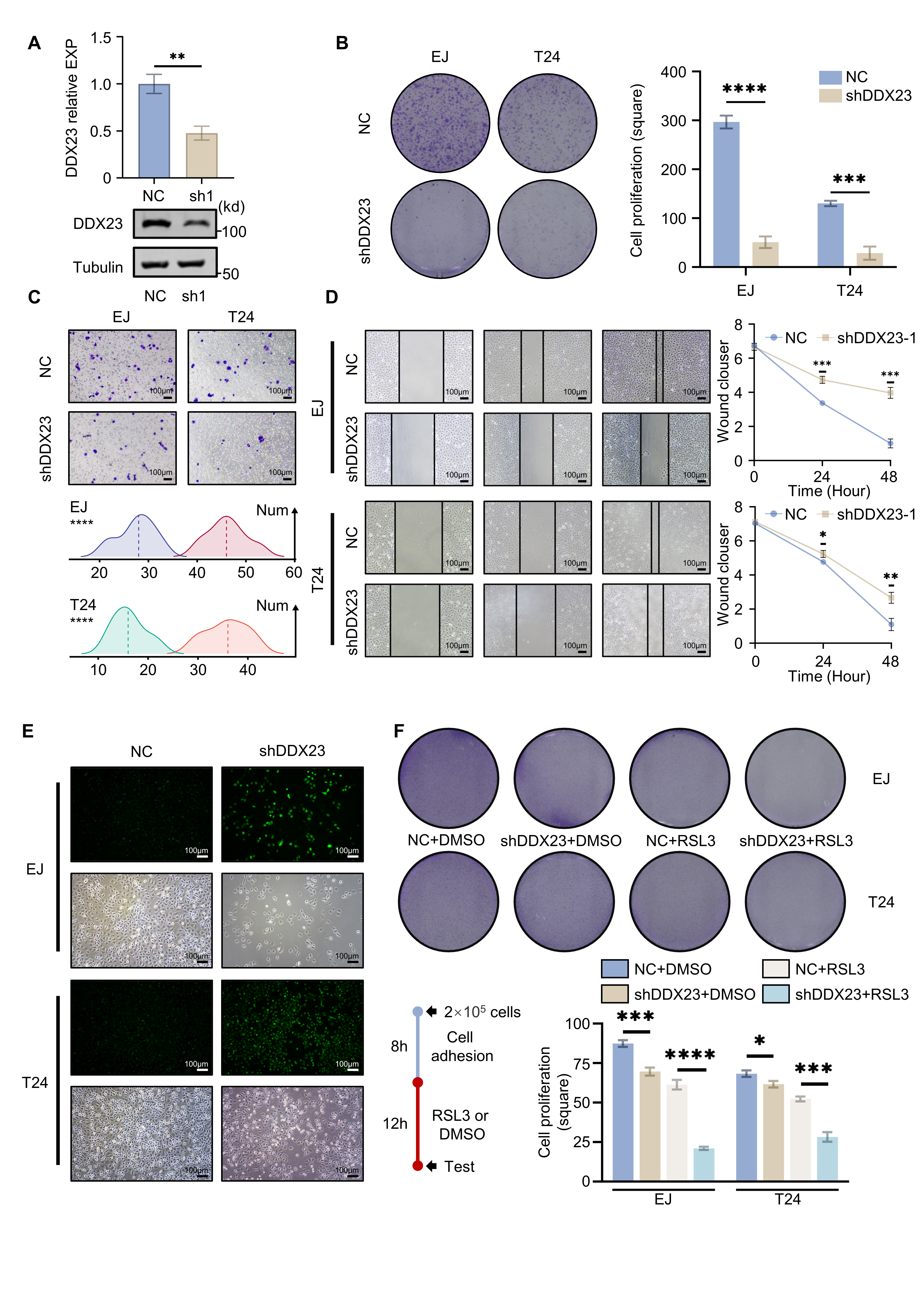


**Supplementary Figure 5.**

(A) Knockdown of PEDS1 in T24 cells. (B) Colony formation assay comparing control and DDX23 knockdown groups. (C) Transwell assay comparing control and DDX23 knockdown groups. Scale bar, 100 μm. (D) Wound-healing assay comparing control and DDX23 knockdown groups. Scale bar, 100 μm. (E) Differences in ROS accumulation between control and DDX23 knockdown groups. Scale bar, 100 μm. (F) Sensitivity to RSL3 (6 µM) in the DDX23 knockdown group and the control group. (Data are shown as the mean ± SD, n = 3. *p* ≤ 0.05 as *, *p* ≤ 0.01 as **, *p* ≤ 0.001 as ***, *p* ≤ 0.0001 as ****)


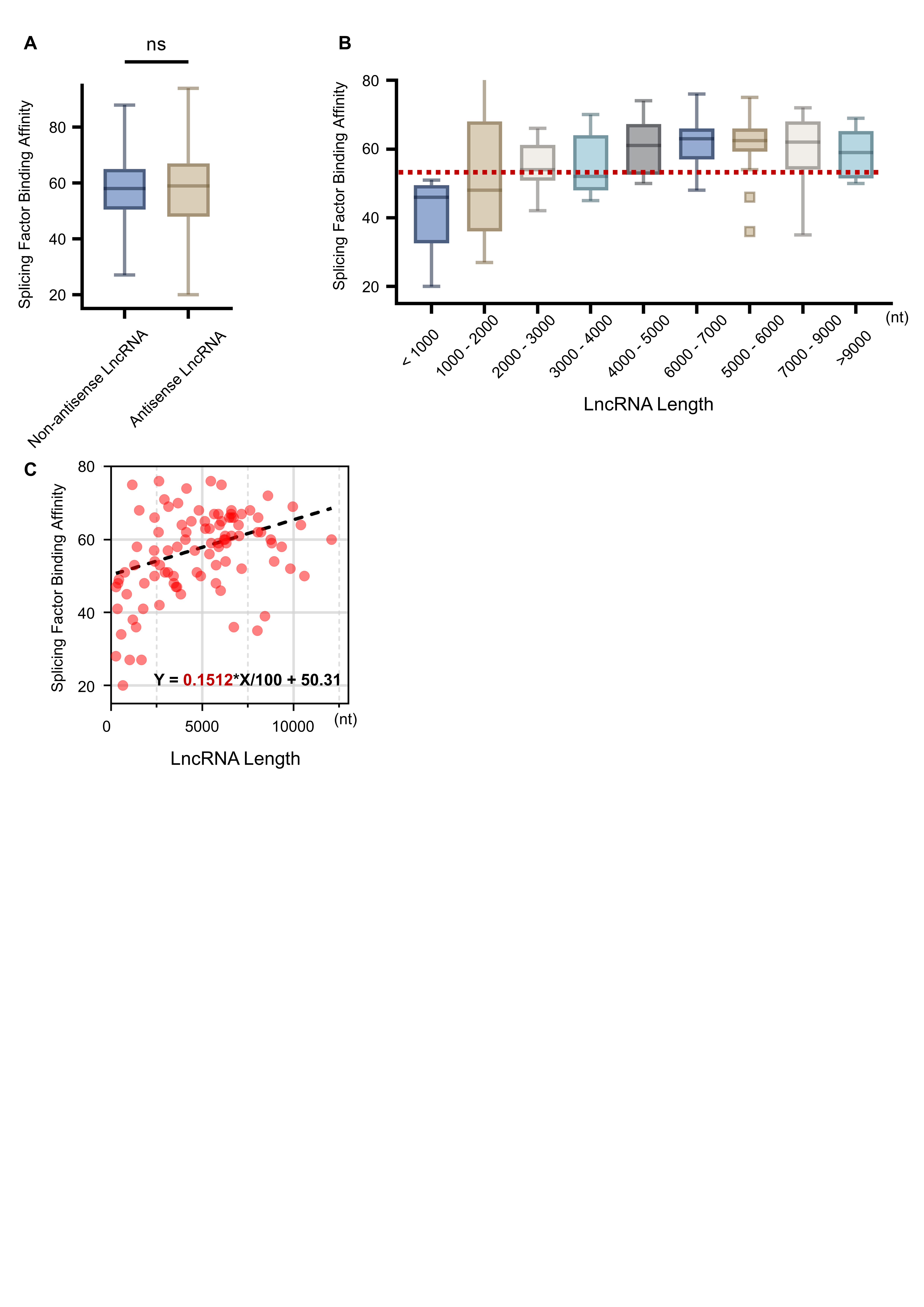


**Supplementary Figure 6.**

(A) Binding strength of splicing factors to antisense and non-antisense lncRNAs. (B–C) Binding strength of splicing factors to lncRNAs of different lengths (n = 100). (*p* > 0.05 as ns)


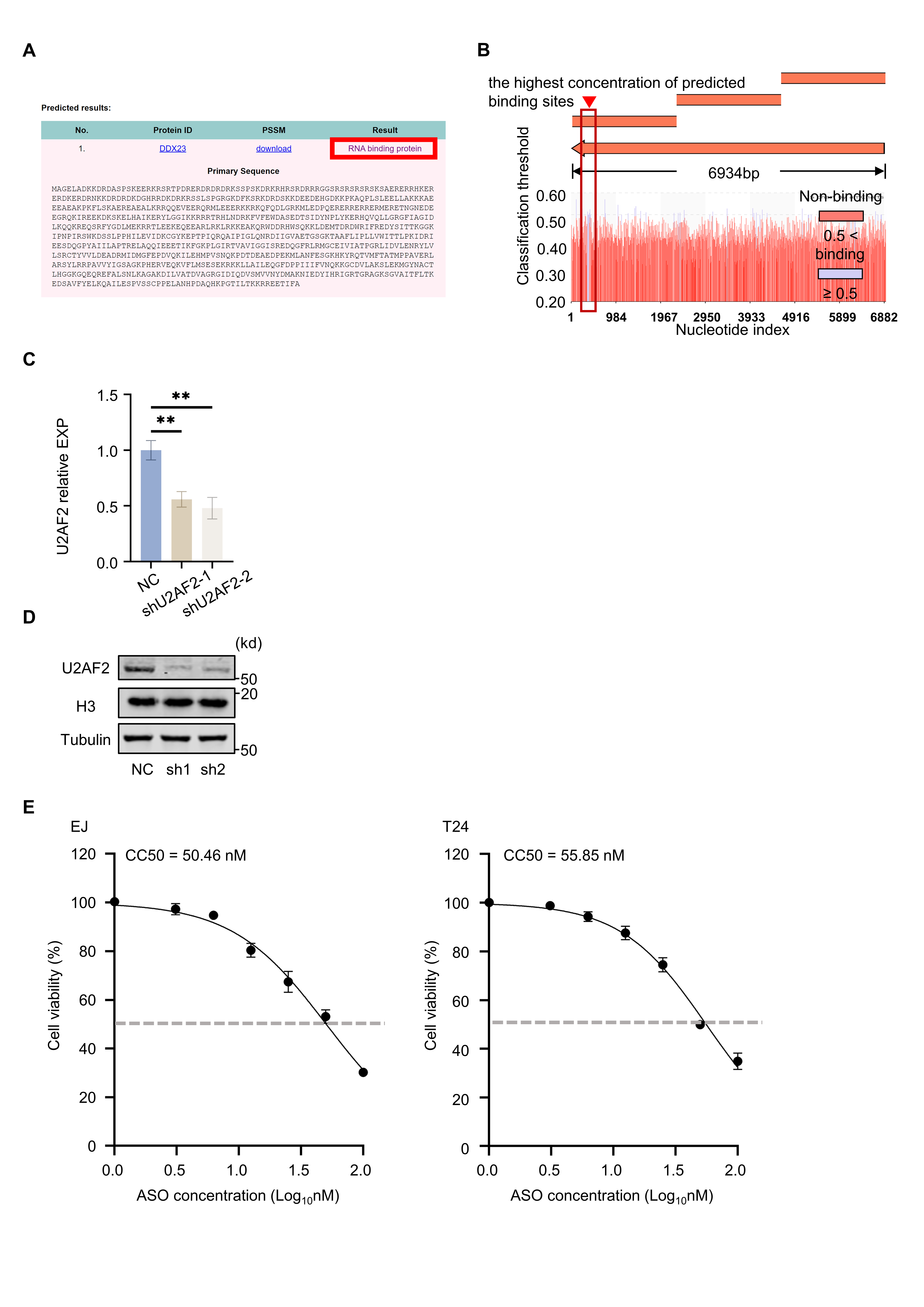


**Supplementary Figure 7.**

(A) Predicted RNA-binding ability of DDX23. (B) Analysis of the binding potential between the primary structure of LncPEDS1-AS and the DDX23 protein. (C-D) Changes in U2AF2 RNA and protein levels in EJ cells following U2AF2 knockdown. (E) CC50 values of ASO in EJ and T24 cells. (Data are shown as the mean ± SD, n = 3. *p* ≤ 0.01 as **, *p* ≤ 0.001 as ***)


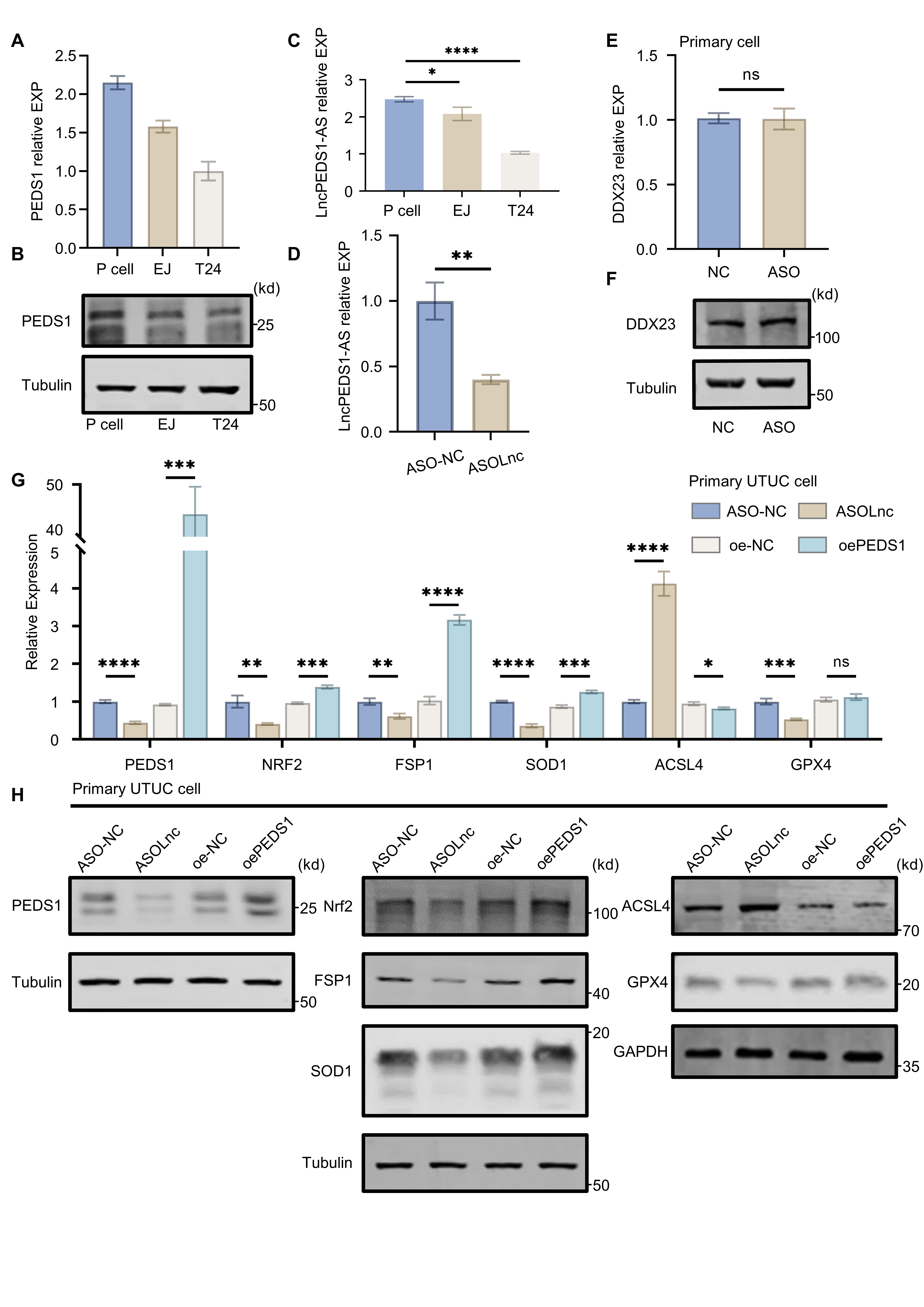
**Supplementary Figure 8.**

(A-C) Relative expression levels of LncPEDS1-AS and PEDS1 in primary UTUC cells versus EJ and T24 cell lines. (D) Effects of ASO drugs on LncPEDS1-AS expression in primary UTUC cells. (D-E) Effects of ASO drugs on DDX23 expression in primary UTUC cells. (F-G) Alterations in key molecules across multiple antioxidant pathways in primary UTUC cells with ASOLnc or PEDS1 overexpression. (Data are shown as the mean ± SD, n = 3. *p* > 0.05 as ns, *p* ≤ 0.05 as *, *p* ≤ 0.01 as **, *p* ≤ 0.001 as ***, *p* ≤ 0.0001 as ****)


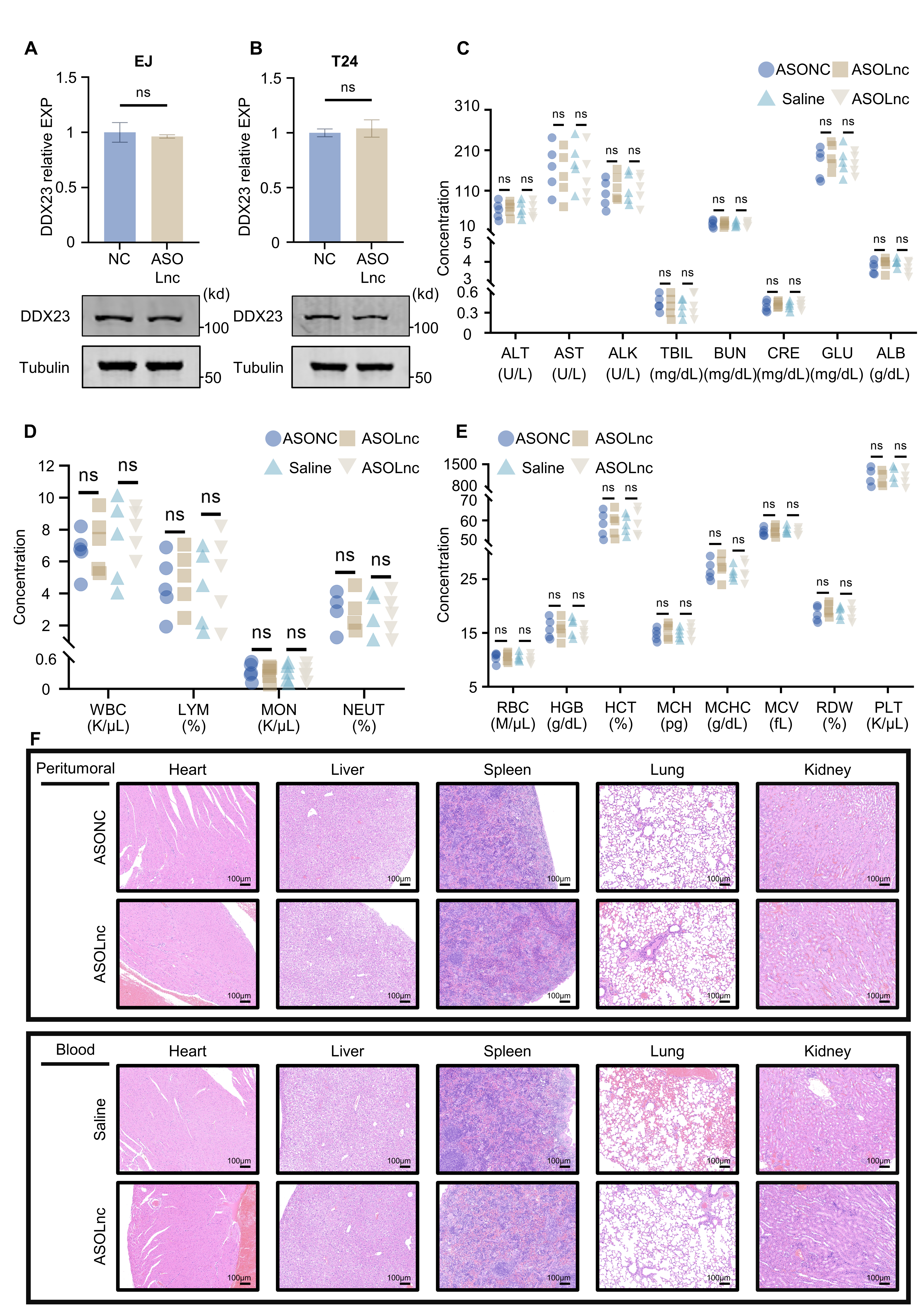
 **Supplementary Figure 9.**

(A-B) Effect of ASO treatment on DDX23 expression levels. (C-F) Haematological and Biochemical Parameters, and H&E Staining of Major Organs in Mice from Different Treatment Groups (n = 5). Scale bar, 100 μm. (Data are shown as the mean ± SD, n = 3. *p* > 0.05 as ns).
