# Supplementary material for: LncPEDS1-AS promotes UTUC resistance to lipid peroxidation by regulating PEDS1 expression via DDX23
Source: Cell Death Dis. 2025 Dec 8;17(1):87. doi: 10.1038/s41419-025-08293-6 (PMC12830725; doi:10.1038/s41419-025-08293-6)
Supplement: Supplementary file 2 — Supplementary Table [file 41419_2025_8293_MOESM2_ESM.docx]

Table S1

| Gene name | Orientation | Sequence |
| --- | --- | --- |
| LncPEDS1-AS | FORWARD | CTCCTCCCTCTTCCACTGCTCTG |
| LncPEDS1-AS | REVERSE | CCTTTGCTTGCTTGGTGTGTTTCC |
| PEDS1 | FORWARD | GGACAACTGCCTGGTGACACTG |
| PEDS1 | REVERSE | CTGGTTGGTGAAGGTGCCGAAG |
| pre-PEDS1 | FORWARD | GCCTTGACCTTCCAGCCTTTACC |
| pre-PEDS1 | REVERSE | GCACAGGATCACAGAGCACCAC |
| LncPEDS1-AS-F1* | FORWARD | CTCCTCCCTCTTCCACTGCTCTG |
| LncPEDS1-AS-F1* | REVERSE | CCTTTGCTTGCTTGGTGTGTTTCC |
| LncPEDS1-AS-F2 | FORWARD | GAGATGGAAGGCGTGGCTGAG |
| LncPEDS1-AS-F2 | REVERSE | CCTGTGGCTGGAAGTGGAGTC |
| LncPEDS1-AS-F3 | FORWARD | GGTTCATCTCTCTCCCCTCCAG |
| LncPEDS1-AS-F3 | REVERSE | CGTTCTAGCCACCTAAGCCAATAG |
| GPX4 | FORWARD | AGAGATCAAAGAGTTCGCCGC |
| GPX4 | REVERSE | TCTTCATCCACTTCCACAGCG |
| NRF2 | FORWARD | TCCAGTCAGAAACCAGTGGAT |
| NRF2 | REVERSE | GAATGTCTGCGCCAAAAGCTG |
| FSP1 | FORWARD | AGTAGTGGGGATAGACCTGAAGA |
| FSP1 | REVERSE | CCACCACGATGAACCGTGA |
| SOD1 | FORWARD | GGAAGTCGTTTGGCTTGTGG |
| SOD1 | REVERSE | GGGCCTCAGACTACATCCAAG |
| ACSL4 | FORWARD | CATCCCTGGAGCAGATACTCT |
| ACSL4 | REVERSE | TCACTTAGGATTTCCCTGGTCC |
| DDX23 | FORWARD | GAGGACTACAGCATCACCACCAAAG |
| DDX23 | REVERSE | GCCAGTCTCAGCCACACCAATG |
| U2AF2 | FORWARD | CGGCAGCTCAACGAGAATAAA |
| U2AF2 | REVERSE | GGGAACGAATCAGTCCACCG |
| ACTB | FORWARD | CAGATGTGGATCAGCAAGCAGGAG |
| ACTB | REVERSE | CGCAACTAAGTCATAGTCCGCCTAG |

* Same with LncPEDS1-AS

Table S2

| **REAGENT or RESOURCE** | **SOURCE** | **IDENTIFIER** |
| --- | --- | --- |
| **Cell Lines** | |  |
| Human: T24 | ATCC | HTB-4 |
| Human: EJ | PROCELL | EJ-1 |
| **Antibodies** |  |  |
| Rabbit polyclonal anti-PEDS1 | ActivAb | Cat#K005799P |
| Rabbit monoclonal anti-PMP70 | Abcam | Cat# ab85550 |
| Rabbit monoclonal anti-DDX23 | Abcam | Cat# OAGA04957 |
| Rabbit polyclonal anti-GFP | Abbkine | Cat# ABT2020 |
| Rabbit monoclonal anti-H3 | Abbkine | Cat# ABL1070 |
| Rabbit polyclonal anti-U2AF2 | SantaCruz | Cat# sc-53942 |
| Rabbit polyclonal anti-NRF2 | Proteintech | Cat# 16396-1-AP |
| Rabbit monoclonal anti-ACSL4 | Epizyme | Cat# 99M02K23 |
| Rabbit polyclonal anti-FSP1 | Proteintech | Cat# 20886-1-AP |
| Rabbit polyclonal anti-GPX4 | Proteintech | Cat# 30388-1-AP |
| Rabbit monoclonal anti-SOD1 | Epizyme | Cat# 33L42M95 |
| Rabbit polyclonal anti-GAPDH | Proteintech | Cat# 60004-1-Ig |
| Rabbit monoclonal anti-Tubulin | CST | Cat# 2128 |
|  |  |  |
| Dylight 800, Goat Anti-Rabbit IgG | Abbkine | Cat# A23920 |
| Dylight 800, Goat Anti-Mouse IgG | Abbkine | Cat# A23910 |
| **Bacterial and Virus Strains** |  |  |
| E. coli DH5α | Thermo Fisher | Cat#18258012 |
| *pMS2-GFP | Addgene | Cat#27121 |
| **Chemicals** |  |  |
| RSL3 | Sparkjade | Cat# SJ-MX0040 |
| Apocynin | Targetmol | Cat# T6391 |
| DMSO | solarbio | Cat# D8371 |

Table S3

| Name | Sequence |
| --- | --- |
| sh-LncPEDS1-AS#1 | CCGGGCATTTGCCATTCCCACTACTCGAGTGAGTGGGAATGGCAATGCTTTTTG |
| sh-LncPEDS1-AS#2 | CCGGCCAACTCACAACCCAAACACTCGAGTGTTTGGGTTGTGAGTTGGTTTTTG |
| sh-LncPEDS1-AS#3 | CCGGGAAGTGAAAGCCTTGATCACTCGAGTGATCAAGGCTTTCACTTCTTTTTG |
| sh-PEDS1#1 | CACCGCATCACCACAGGCTGGCTCACTCGAGTGAGCCAGCCTGTGGTGATGC |
| sh-PEDS1#2 | CACCTGACATGAAATGGGCCCAGAACTCGAGTTCTGGGCCCATTTCATGACA |
| sh-DDX23#1 | CACCGGAAGGACAGAGACTCTAAGACGAATCTTAGAGTCTCTGTCCTTCC |
| sh-DDX23#2 | CACCGGATGAAGAGGATGAACATGGCGAACCATGTTCATCCTCTTCATCC |
| sh-U2AF2#1 | CACCCGACGAGGAGTATGAGGAGATCTCGAGATCTCCTCATACTCCTCGTCG |
| sh-U2AF2#1 | CACCTGCAGATTAACCAGGACAAGATTCAAGAGATCTTGTCCTGGTTAATCTGCA |
| sh-NC | CCGGTTCTCCGAACGTGTCACGTCTCGAGACGTGACACGTTCGGAGAATTTTTG |
| ASO | TCCAGAGGAGGGTAAGAACT/ CAGATGATGTCAACTCCCTG/  CCATTCCGGAAACACACCAA/ |

| MS2✖6 Loop |
| --- |
| acatgaggatcacccatgtctgcaggtcgactctagaaaacatgaggatcacccatgtctgcagtattcccgggttcattagatcctaaggtacctaattgcctagaaaacatgaggatcacccatgtctgcaggtcgactctagaaaacatgaggatcacccatgtctgcagtattcccgggttcattagatcctaaggtacctaattgcctagaaaacatgaggatcacccatgtctgcaggtcgactccagaaaacatgaggatcacccatgtctgcagtattcccgggttcattagatcctaaggtacctaattgcctagaaa |

Table S4

*pMS2-GFP Addgene Cat#27121

Table S5

| **REAGENT *** | **SOURCE** | **IDENTIFIER** |
| --- | --- | --- |
| Advanced DMEM/F12 | Thermo Fisher | 12634010 |
| Y-27632 | Targetmol | 146986-50-7 |
| Pen–Strep dual antibiotics | Procell | PB180120 |
| Amphotericin B | Sigma | V900919 |
| GlutaMAX™ Supplement | Thermo Fisher | 35050061 |
| B-27™ Supplement | Gibco | 12587010 |
| N-Acetylcysteine | Sigma | A9165 |
| Nicotinamide | Sigma | N0636 |
| A83-01 | Sigma | SML0788 |
| HEPES | Sigma | H4034 |
| Noggin** | Novoprotein | CB89 |
| Wnt3a** | Novoprotein | C18K |

* The recipe was adapted from that described in “Patient‐Derived Upper Tract Urothelial Carcinoma Organoids as a Platform for Drug Screening” and subsequently refined.

**Reagent concentrations were taken from the above Reference *, with the following modifications: Wnt3a at 100 ng/mL and Noggin at 100 ng/ml.

The principal motivation for these modifications was our observation that, under the revised formulation, UTUC organoids grew more rapidly and displayed enhanced spheroid-forming capacity. (**The process was arduous and often frustrating, marked by numerous failures**)
