# Supplementary material for: LncPEDS1-AS promotes UTUC resistance to lipid peroxidation by regulating PEDS1 expression via DDX23
Source: Cell Death Dis. 2025 Dec 8;17(1):87. doi: 10.1038/s41419-025-08293-6 (PMC12830725; doi:10.1038/s41419-025-08293-6)
Supplement: Supplementary file 3 — Dataset 1 [file 41419_2025_8293_MOESM3_ESM.docx]

GGCAGCGGGAGTTTGAATGGGAGAGAGTAGTCAAGGGGACATTGGAAGAGAGCTGACCTTCACTGGGCTGGAGGGGCCCAATGAGGAGTGAGTCAGTACAGGATAGGACACAGGGACCGGCCAAGGAACTGGAAGTGGGGGCCTCCCACCAATGGGCCTGGGGCCACTCAGGCCTCCAACCCCCATATTCCTCTACTTTCTTACTATATACCAGGCACTTACTTATATTTGAGCACTTACTATATACCAGGCACTGTGGTGGCCTTATCACTCAGCTCAAATGCTACCTCTGCCAGGAAGCCTTCCCTGATCATCCACTTAAGCAGCACCCCAGGTTTCTCACATTACCCTTTATCTATCTATCTATCTATCTATCTATCTATCTATCTATCTATCTATCATTTATCTATCTATTTATTTGAGACAGAGTCTCACTCTGTGGCCAGGCTGGAGTGCAGTGGGGCGATCTCGGCTCACTGCAACCTCCACCTCCCGGGTTCAAGTGACTCTCCTGCCTCAGCCTCCGAGTAGCTGAGACTACAGGTGCACACCACCATGCCCAGCTAAGTTTTGTATTTTTAGTAGAGACTAGGTTTCACCATGTTGGCCAGGATGGTCTCGATCTCTTGACCTCGTGATCCACCCGCCTCGGCCTCCCAAACTGCTGGGATTACAGGCATGAACCACTGAGCCTGGCTCTATTTACAATTTTTTTAATGCAGATGGGGTCTTGTCTTATATAGGCTGGTCTTGAACTCCTGGGCTCAAGCAATCCTCTCGCCTTGGCTTCCTAAAGTGCTGGGATTACACGCGTGAACCACCATGACAGGCCACCCTCTTTCATTTCTTTATAGCCTTTATGATAACCTGTAACCATCTTCGTTAATTCTCTATTTTCCTGTTCTGTGTTTGTTCACCCCCCAATCCCCAACTGGAACGTTATCCCCAAAATGCAAAACACCATGTTCTTTCACTGCTGATCCTCAGTGCTTAGAATACAGCCAGGCGCAGAGTAGGTGCTGAATAAATATTGCTCACAATCTTACAGCCTTCCAGCTCACCATGAGGTAGATACTAATATCAGTCCCATTTTCCACATGAGCACACTGAGGCTTAGAGAAGAGTTTCCCAATTAGCAGGTGGCCAAACCAGGATTGGAACTCAGCCCTGCCTAAGTAGTTAACCCATCTCCTAAATAGCCTGAATTCTAATCCCTCCACCTGCCTTCCAAGGAGCACAAATTGAGGACATGAAAAAGAATGTTTTTTTGTGGTTCTCAGTTCTGTTTCCAGAAAGACCTAAAAAAAAATAGTAGCTGTAATTTATCAAGTACCTACTATATGCCAAACACCGCATTTGCCATTCCCACTATGGCCCTAAGAAGGTGATATATGAAGGAGGCGGCTGCAGCCCTATTTTGTAAGGAGAAAAGCGGTCCCCAGGGAGGTTTGTGAGTTGCCTAAAGGCACATGTGGAGAAGCTGAGAGTTAGGCCTGAGTTAGACTTGACAGCACCTGATACACTTAACCTTGAAAGCAGCCCAGCTGGCTCCAGAGAAACTCCCAGTTCACCTCACCTCTTTCGCACTGGCTGCTAACTGAGCATGGCACGTGGGCCTCCTTTCCCTTCCTCCACTGCACTGGGCATGTTCCTGCCTCTGGCCTGCTGCTTCCTGTTACCTTTGCTCTAATCTGTGCTCATCTATCTTCAGGGCCCTCCCACACTTCTCTCCTCCCTCTTCAGTTGCTCTGCCCCCTTCTCTCCTCCCTCTTCCACTGCTCTGCCCGCTTCTCCCTCTTCCATTGCTCTGCCTCAACTCCAGTCAGGAGCCTCTTTGCCATTCCGGAAACACACCAAGCAAGCAAAGGCCAGCCTCAGGGCCTTCGCACCTACTGTTCCTGCTGCCTAGAGTGCCTCCCACAGAGGTCTGCACGGCTTTGTCCCTCAGCTCCTCGGGGTCTTTGTTCAAGTGTCACCTTCCCTGACCACCCGATTTAAGAGGCAAAGCCCCCACTTCCTTCTCCCACCCCTGCTTTATTTTCCTCCATAGCAGTTAGCACCATTAGCCACGCTACACATTTTATTCATTCGTTCCAGCTCCCTCACTAGATGGTGAGTGCCACAAGGGCAAGGATCCGTGTTTTCTGGTTTTTTTTTTTTTTTTTTTTTTTTTGAGACAGTCTCTGTCACCTAGGCTGGAGTGCAACTCCGTCTCAAAAAACAAACAAACAAAAAAAACTGGACAAGAGGAGCCTAAAGGGACATGACAACTAAAGGCCATGTGATACCCTGGATGGGATCCAGGAACAGAAGAAGGACATTAGGGAAGAACTAAGGCAATCTGAATAAGCCATGGACTCCAGCTCATGATAATGTATCAACACTGGCACACACTGTAACAAATGTGCCCCGCTAATGTAAGATTTAGGATGTGAATAATGGAGGAAACTGCGTGCCCCAGGCATATGGGGACTCTCTGTACTATCTGCCCCATCCTTCTGTAAGTTGAAAACTTGTTCTAAAAAATAAAGTCTGTTTTTCAAAAAGTGGACAATCTCACTTCATGTCCACAGTAACAGGCCCCACCCCATCCTGACCTTCCAGAGGAGGGTAAGAACTCCCCAAGGTCACACGGTGGCAGGGGCAAGGCAGGGCTGCTGGTGAGTAGCCAGAGACTCGCTAGAGTCATTCAGGATGATGCAACCTTAGGGTTAGGGTTAGGGAGACTTCGCACCCAGGTATGGAGAATGGGGACATTTACCTCCCTTAACTATGAGAAGTTAATAAAATGGGCTGCTGGGTAAAGGGCCCGGTGGGTCCCACGACACAGCAAGCATCCCCCCAACGCAGCTCCTGTGAGCTCTCTGCTGAGCCTCAGTCTTGCCATCTGTCCAATGGTCAAGGCCTGGCGCCTGCACGCTGCCCAGCAGGGAGGGATGTCTGCTTTGGACAGAAGTCAGGAATCATCTTAGGAGACCAGGAGCCCTCCCCCTGGCTCCGTGATCTTGCGGCCTCTGCCGGTGGCAGTGACTCCAGGCTGCTGTCTGAGGCTGCTTTAATGGGGGCAGGGGTGGCAGACAGGCACACAGAAGCCTCTTTGTCCCAGCTTTGGGGTGGTGGCAGCTGGCAGCCGGCTCCCCCTGGGGGCAGCTTCGGCTTGGTTACCACCCTTCCCCGCAGAACACTGGGACAAGGAGAGGGATGGGAGACGGCTCACCCCAGGCTACCCTGACTCACGGGGGAACTTGAGGTAGTCGCTGGGCCCGTGCGGGCTTCCATTTCCCCAGAGGTCACCCAGCTGGGACTTCCTCACCCCTCCCTGCCCACCTCAAAGGCCTGGCGTGGAGATGGAAGGCGTGGCTGAGGAGCCAAGTGAATGCACTGAGGGCAAATCCATGGGTTTCGGTCCTGGGTCCCAGTCCTGTCTCTACCGCCTTCCAGCTCAGTCAGATCCAGACTCCACTTCCAGCCACAGGACGGGCCACGACCCGAGCCTGGGACTTCTCTGACCTCATCCCTGACCTCTGGACTCACTGTGCCCTCTGCCCGGAAGGCTTCTCCCAAGGTCCACGAAGGACTCTGCTCCTTTAAGGGCCAACTCACAACCCAAACACTTCCTCCCCCTTCTCCACGTGGAACAAAAGCTCAGGGCTCCTACGGCCTTCACTTGTTCACCCACTCACTCACGACAACGATGTGCGTGTCACAGTGGCTGATGACCTCCACGTGGCTAAATCCAAAGGTCCCTTTACTGGCCACCTTGCTGGTCCACTCGGTGGCTGTTCCCAGTCGACCCCCAAGACTCCTGGAAACACATTCTCCTCTTGGCCCCTGCAAGCCCTCCCTGGTTCTCCCCCGACCTCCCTGGTTCTCCCCCCATCTCCCTGGCAGCGCCATCCCAGCCTCCCTTGCTGGCTCTTCCTCCTCCCCAGCCTGGAAACACGGGAGACTCAGCCCCTGGGGGTGCTCAGCCAGCCTAGGAGATAAGGAGCTTCATCTTGACACAGATGACCCCCGGATTTCTTTCTTTTTTTTTTTTTTTTTCCAGAGGAAGTCTCACTCTGTCGCCCAGGCTAGAGTGCAATGGGGCGATCTTGGCTCACTGCAACCTCTGCCTCCCGTGTTCAATCAATTTCTCCTGTCTCAGCCTCCCCAGTAGCTGGGATTATAGGCGCCTACCACCATGCCCAGCTGATTTTTGTATTTTTAGTGGAGACAGGGTTTCACCGTCCCAAAGTGCTGGGATTACAGGTGTGAACCACCATGCCCGGCCGGACCCCCAGATTGCTATTTCCAGCTGCCTCTCCCTGCCTCCAGACCTAGATTTCCAGTTTCCTGCTCAACACCTCTCCTTAGCTCAACAGCGGCCATCTCAAGCATCTCCCTCAGGCCTGCTCTGCCGATGACCTCTCTCCATCTCTGTGAATGACAGCTTCATCCCTTCCATCTCTCAAGAGAGAAACCTTGGAGTCAAGCTAATTCTCTCTCACCTCCACAGCCAACCTGGAAGCACATTCTGGCAGCTCACCTTTCACAGCTCCTCCAGAATTTGACAACGCATCACCACCTCCACCGCTCCACCCAGGTCTGGGCCACCCTTAGGTCTGGGCTGCCATCTTCACGTGGCTGGACCATGGCAGGCGCCTCCTCCCTGGCTGTCCCCACCCCCAGCCATCTTCCCTGCAGCATCAGATGATGTCAACTCCCTGTGGGAAACTCACCAATGGCTTCTCATGACCCCATATCCAGACCAGGCCCTCATGGCCCCACAACATCTGCTTCCAGTGCCTTGCTGGGCTGCCACAATTTTCCTTTGATCAATTTCCTCCAGCCACAATGGCTTCGCTCTGTTCCCCAAACACACGAGGCACGTGCCAACCTCGGGGCCTTTGCATTTGCTCTTCCCTGTGCCTTGGATGCCCCTCCCCAAGATCTCTGCACGGCCCCTCCCTCACCTCCTTCAGGTCTCTGTGCAAACGTCATCTTTTCAGAGAGGCCTTTCCCGCCTACCCGCTTAGCTTGATTTTGCTTCCCAATGCTGCATCACGTGTGTCTGTGTTTATTAACTCGACATCTTTTCGTGCTTCTGCCTTCCTTGATTTCTGGGACCTGCCTGGGACAGTGGAAGCTCTGGGGAGATAGGGGTAGGGGGCAGGCTCTGCAGGCTGGCACAGTGCCCTTACTGCCTGCATGTTAACACACAAATCGGGGTGGCTATTTAAATCGATTAGGGACTGTGCAAACACCATTATCAGGGGATTGTCTGCTCTCATCAGCCCAGAGAGAAATGTGTGGGATGGGGAAGCTGGTGCTGTGCAGCCTCGAAAGCCAGAGATTTCCTGGGCTGTGGCTGGAAGATCAGATCCCAACCAGGGAAGGGAGATAGTGACCTACCTGGGACATCCACCCTCCCAGCCTGCACAGCACACACCTGGGCTCTGATCCAGCCCATCCTTTACCCCCGTAGAGGCCTGAAACAGCCTGGCCTCCCAATGTTAACTGGAGGCTATACACGCCCTGGTGGGGAAGTCCTCAACTGAGAGCCAGGCAGGTCTGGATTTGCAATCTCAGACTCTGCTGCTGCCTGGGTGAGGACCAATATGTGACTTACTTAGCCTCTCAGATGGTGATGGGAACCTCCTCACTAGGGTAGCACAAAGAGTGAAAAGTGCCCAGCATGGGGCTTGGCACATAGTAGGTGCTCACAAAACATCCGTGATTAAACTGACCAGCGAGGCTGAAGTGAAAGCCTTGATCAGTGGTCTGGCAGGTGCTGTCCCCTCCCCGCCCCTGCTTAGTCACTGGTTTTCTCAGCACTTAGGCACACCTGCATTTTGCCTTCACAACAGCCCTAGGAGGCAGGTACTGCCAGCAGCCTCATTTACACTGTGGTAAATGAGGCTGAGAGATGCAATGTGGCCCAAGGCCACAGAGCCAGGAGGTGGTAGAGCTGGGACTTAAACCCAGGTTTGTAGAGGCCTGCTCGTAACCATCCCCAGATGAGGCTGCAAAGCTCAGACTGTATTCCTGCCCTTGAATATGGGGCTTGAGGCAGAGAGGCTGGGGGTGCAGGTCTCTCCCATCCCTGGGGGCAGGGGACATGCCTGGCTCCTGCCTGGGCTTCCCAGCAATGGATGGGTTCATCTCTCTCCCCTCCAGCTGGTCCGGCCCCCGTCCCCAGCATATACCAACCCCACCCCAGACCCAGATGGCTCAATTAGCTGGCCTGAGCACTATTGGCTTAGGTGGCTAGAACGCTTTTAATGAGATTGTGGGAGTTGAACCCTTAGGAAGGGCCAGGCAGGACCAAGGGCACAGCCGCCCCCTTCAGTCTTGGAGCCGGGGATGGGGAGGGGAAGGGTGGTGCATGGAGAGGAAGGAAACTGAGTCATCCAGGGCCTGGGAGTGGAGATAGTGTATTAACAACTATGTTGTACTCTGCCTTGAAGTGTGACTCTGGACTAGTCCCTTCTCTGAGCCTCAGTTCCCTCATCTGGAAAATGATGACAACCTTAGCGTAAGAAGTGACCGATGGAAAACACCAGGAGGTACTAATAAACAACAGCTCTCTCATTTATTCATTCATCAATTCAAAAAATACTGAGCACCGGCTGTGTGCCAGGCCTGAGGCTTGAGCTAGGGACACAGCAGTGAATGAAACAGACAAAAATCTCTGCTCTCAGAGAGCTGGCATTTGAGTTGGGAGAGATGTTGGGAGAGATAGATGATAAACAAATAAAGACACTGTGGACACAGTGTTAACAACAGAAAAGGGGGATAGTGTGTGACAGGCCTGCTATTTTGAGACGGAGTCTCGCTCTGCTGCCTGGGCTGAAGTGCTGTGGCGTGATCTTGGCTCACTGCAACCTCCGCCTCCTGGGTTCAAATGATTCTCCTGCCCCAGCCTCCCAAGTAGCTGGGACCTACAGGCGCC

**F1: Red**

**F2: Blue**

**F3: Black**

***** **This lncRNA is too long to apply RACE technology.**

**** F2 and F3 lack introns on the DNA, meaning F2 and F3 are contiguous, whereas there is an intron between F1 and F2, indicating that F1 and F2F3 are discontinuous on the DNA (Figure 4B).**
