# Supplementary figures and images for: LncPEDS1-AS promotes UTUC resistance to lipid peroxidation by regulating PEDS1 expression via DDX23
Source: Cell Death Dis. 2025 Dec 8;17(1):87. doi: 10.1038/s41419-025-08293-6 (PMC12830725; doi:10.1038/s41419-025-08293-6)

Figure 3

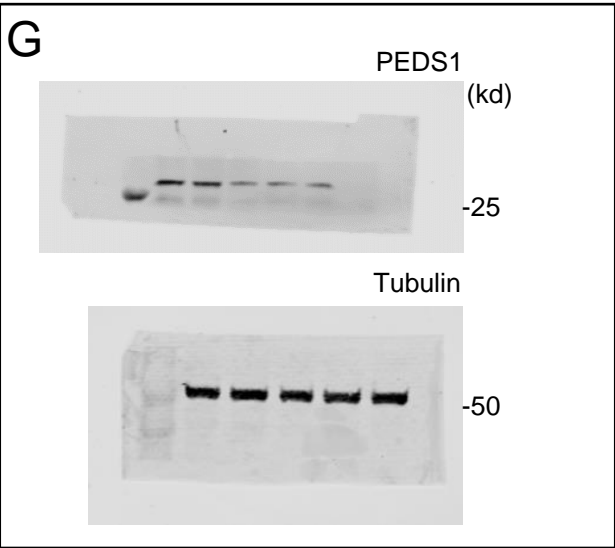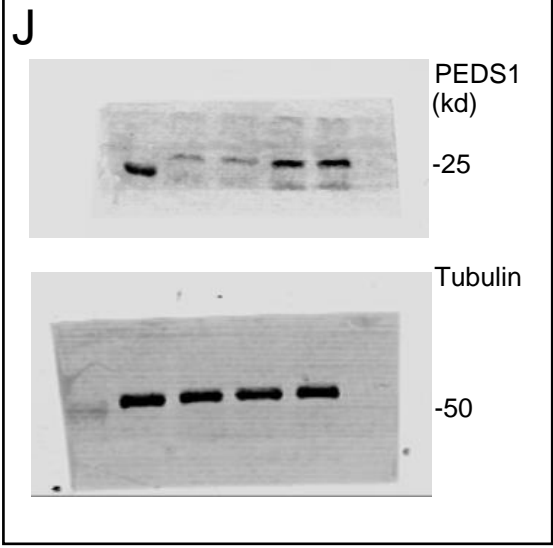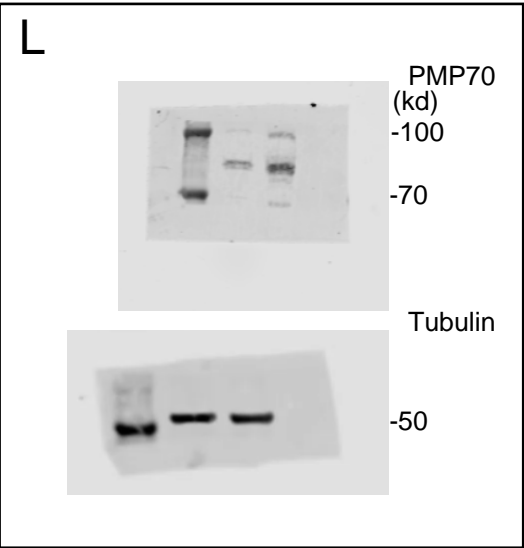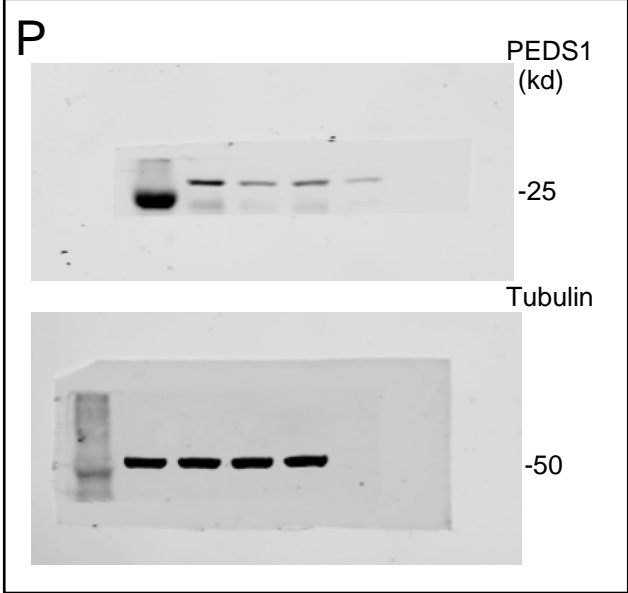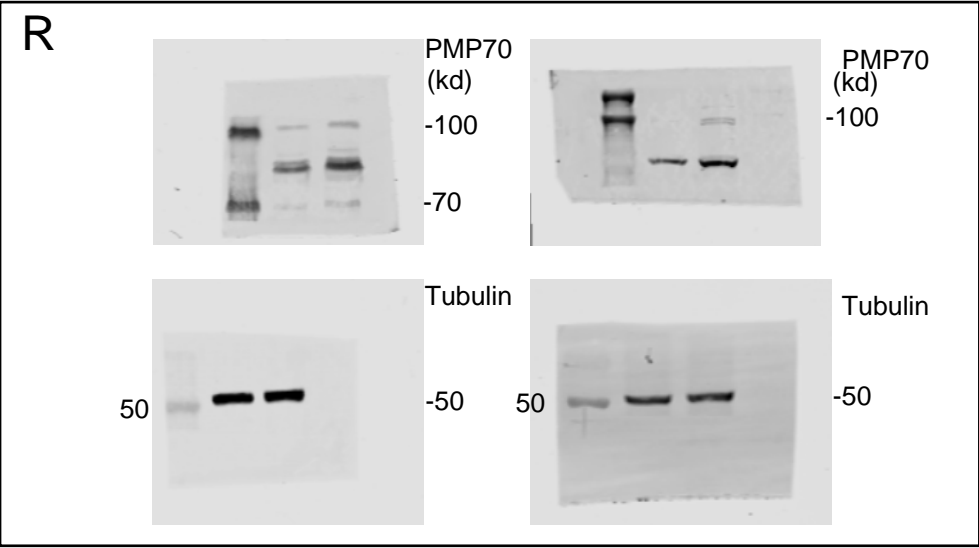

Figure 4

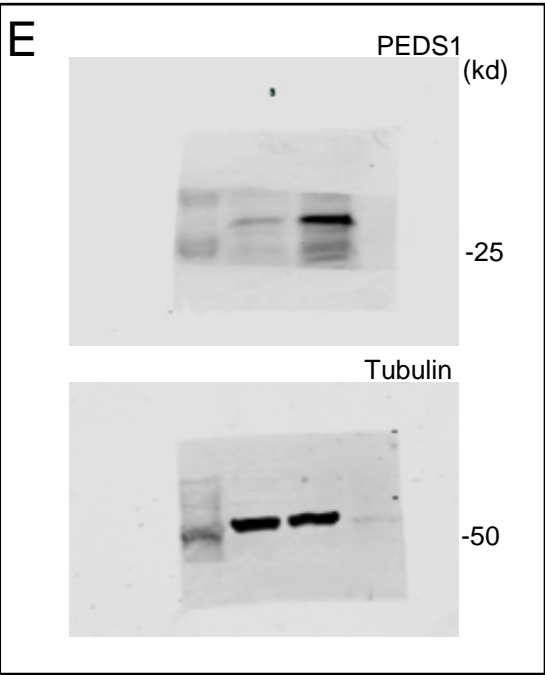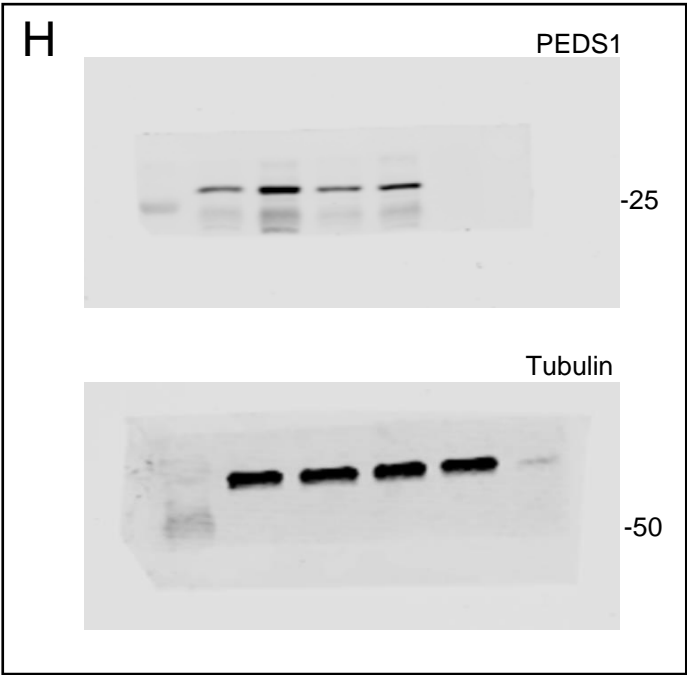

Figure 5

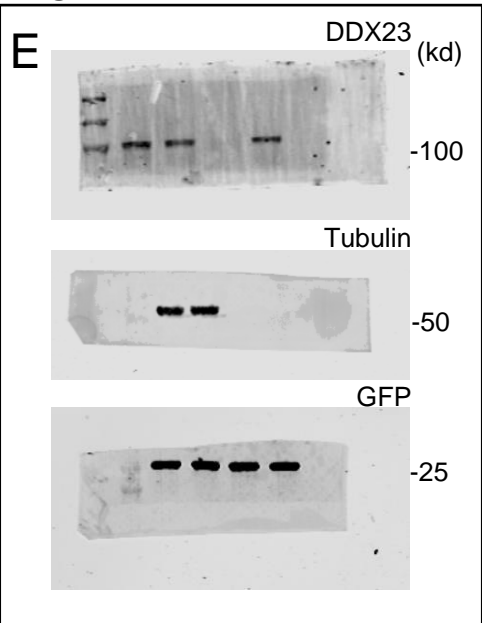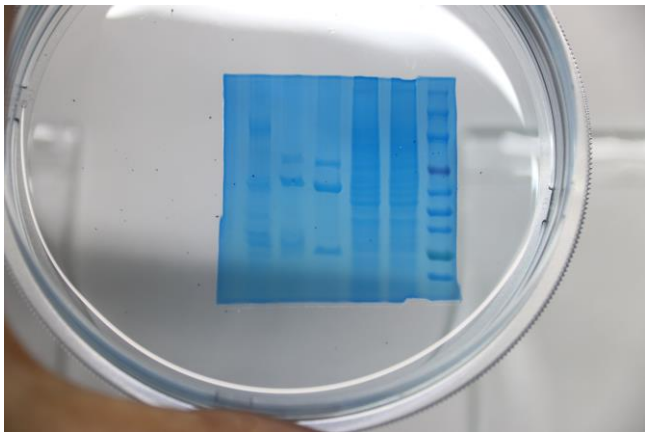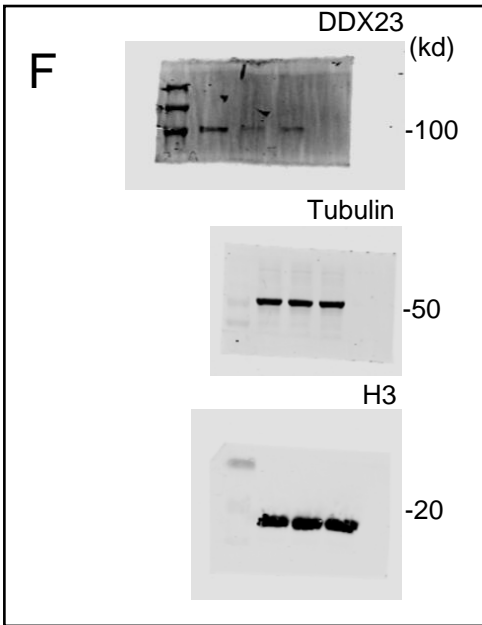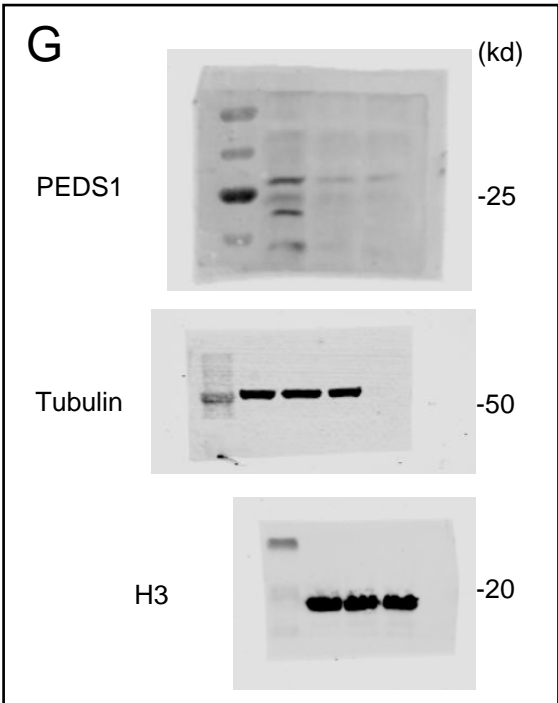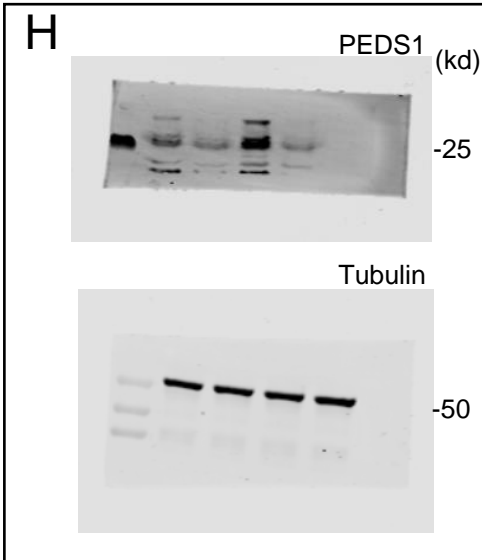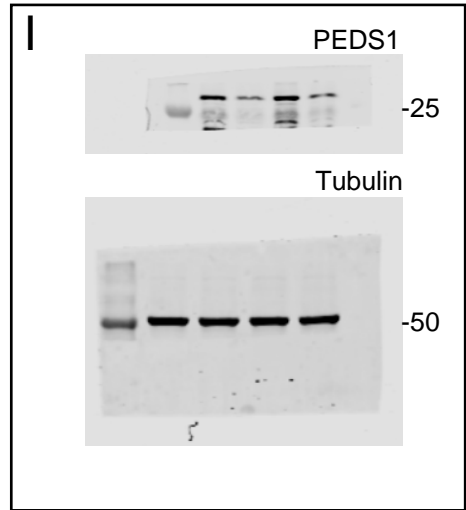

Figure 6

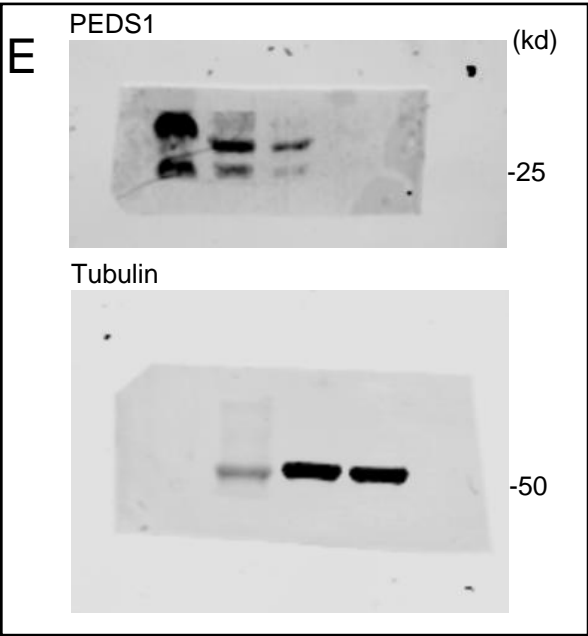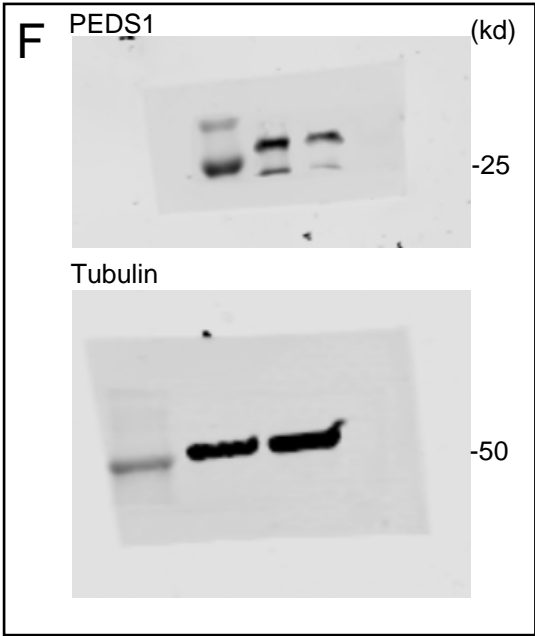

Figure S2

B

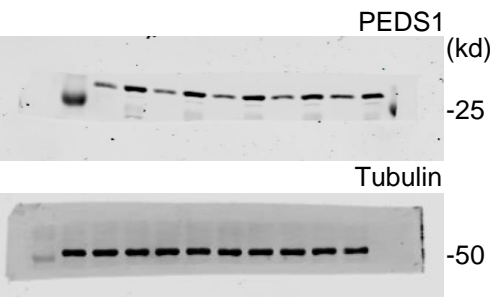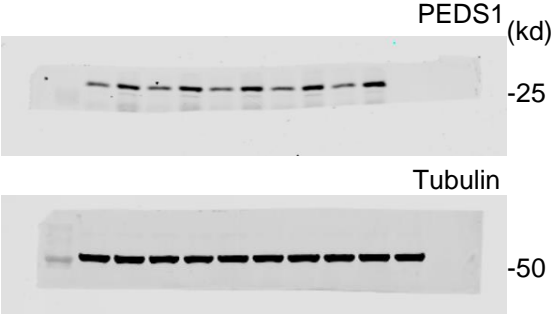

E

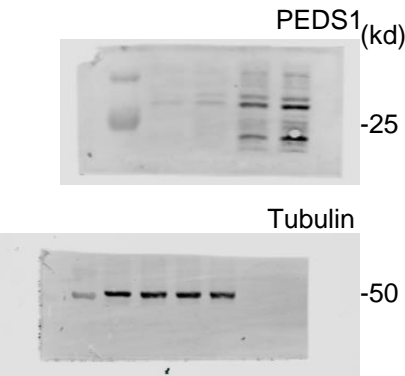

G

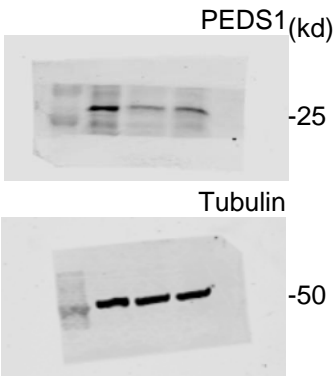

I

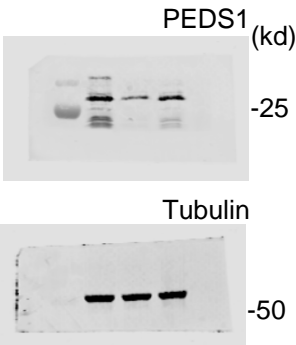

L

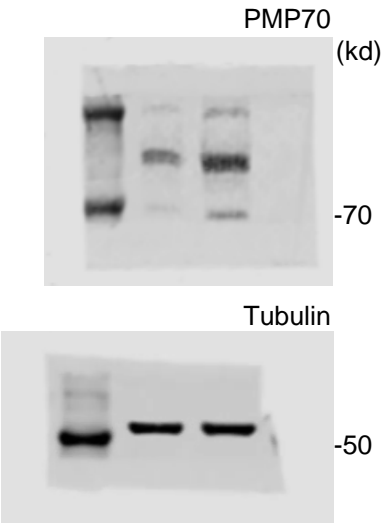

Figure S3

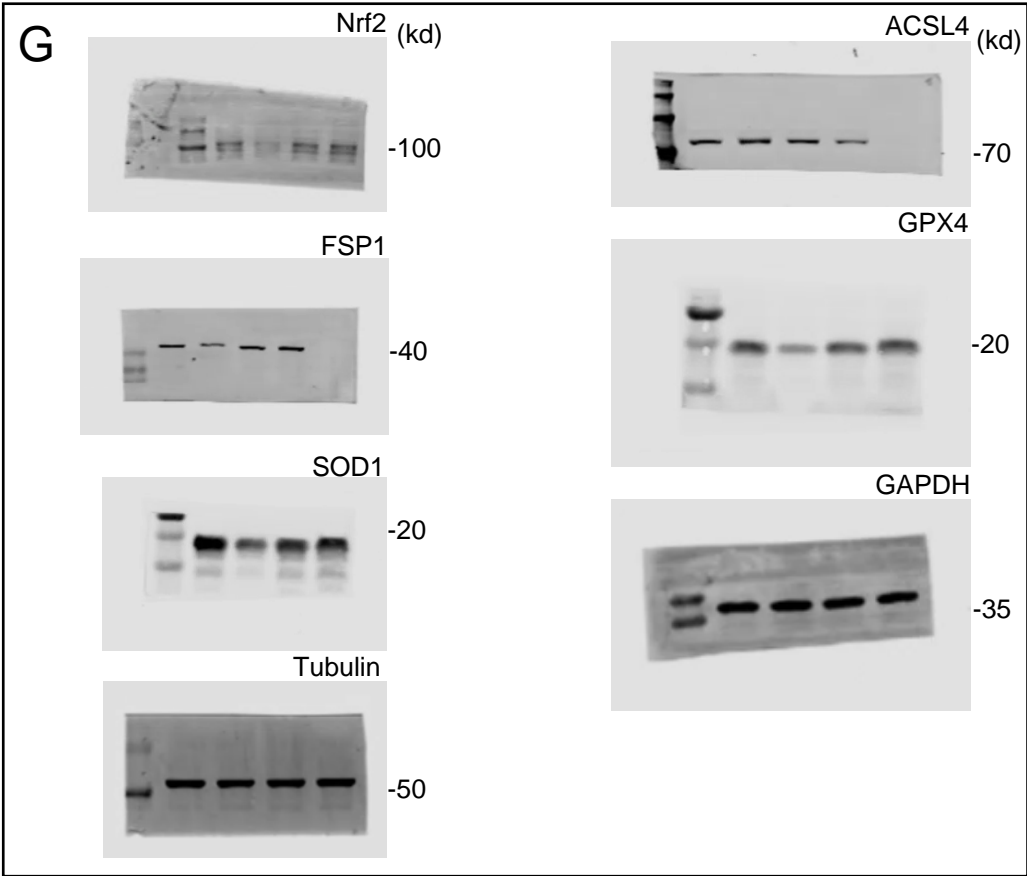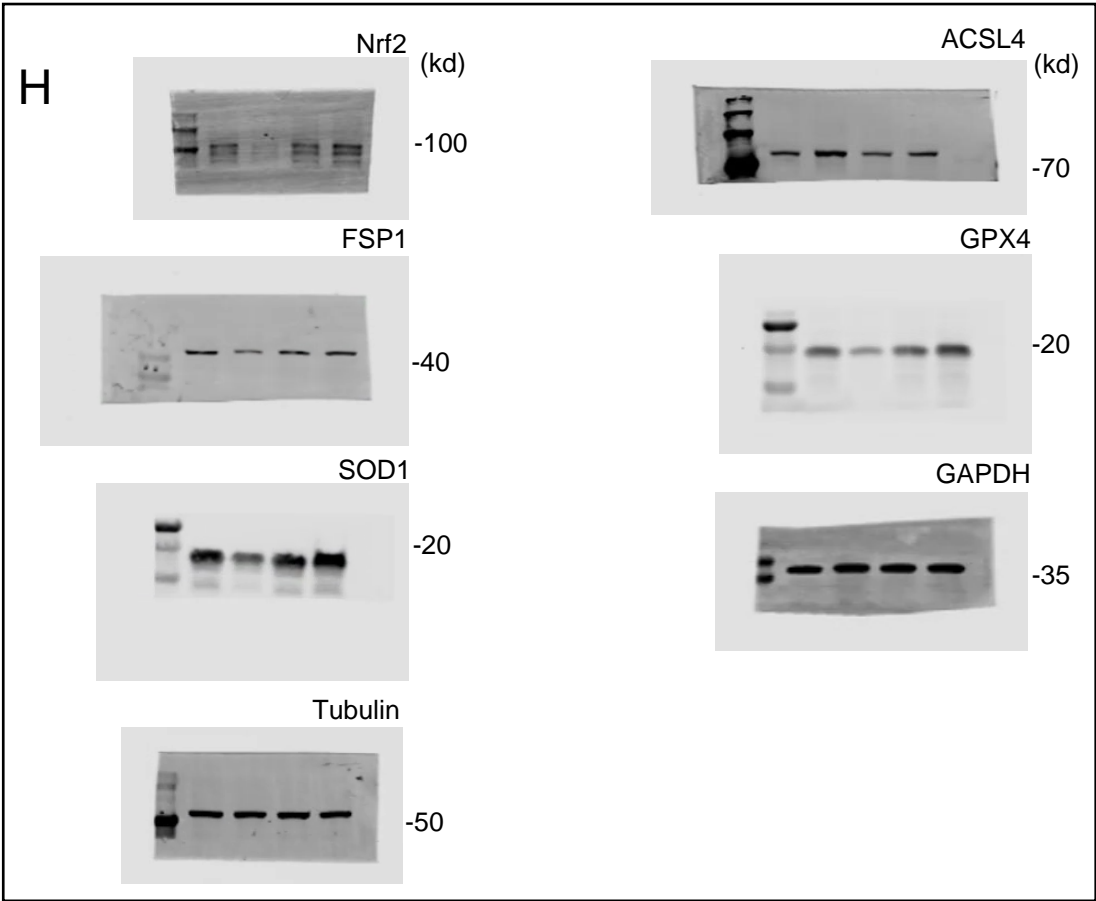

Figure S4, S5, S7

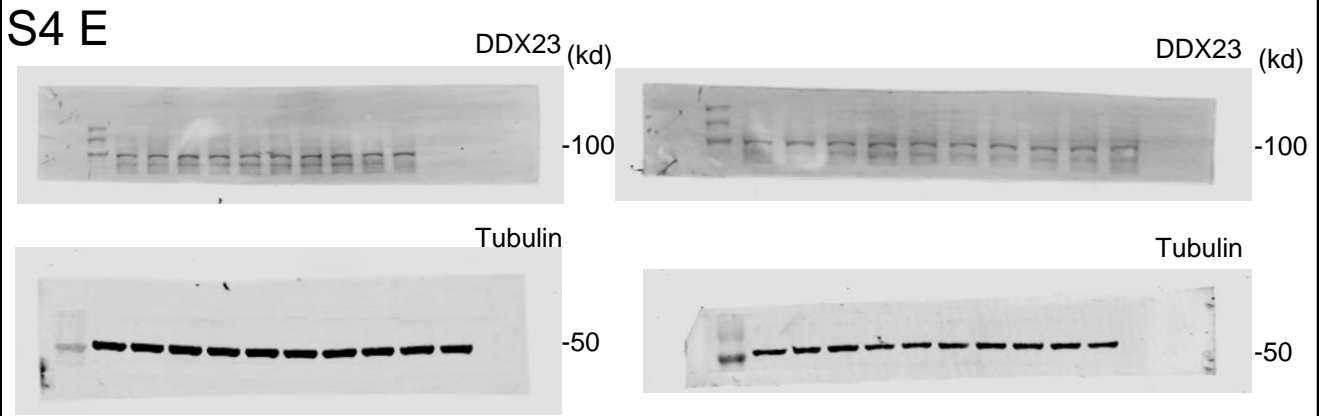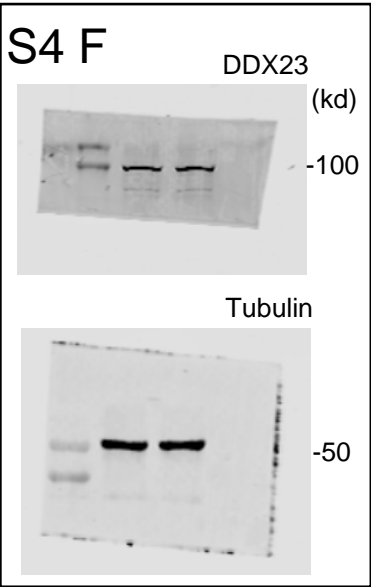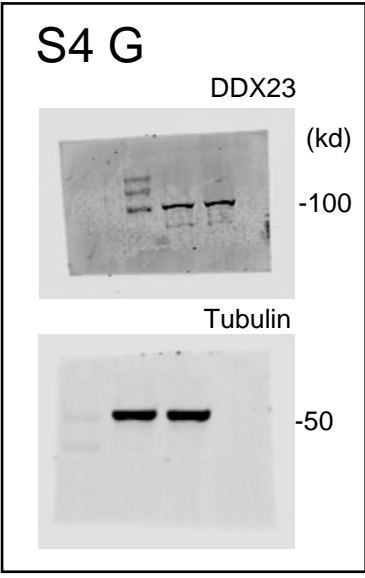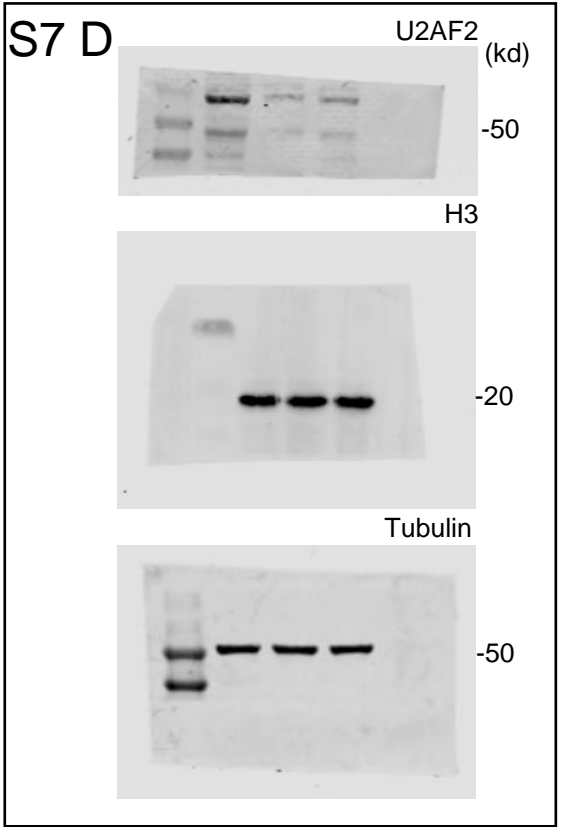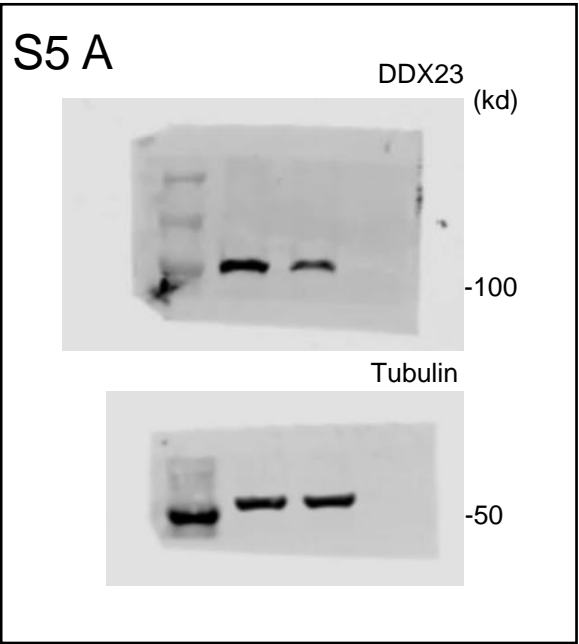

Figure S8, S9

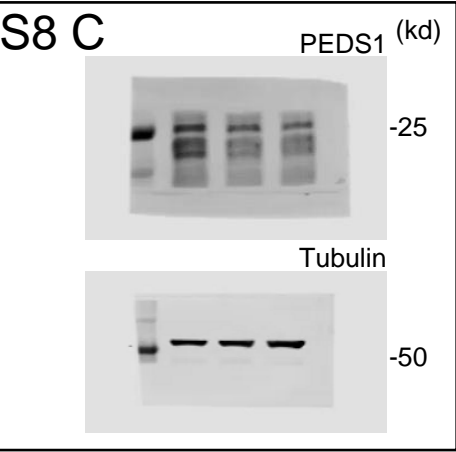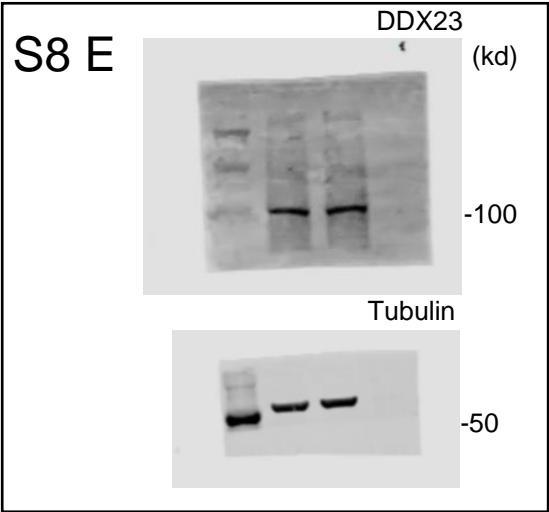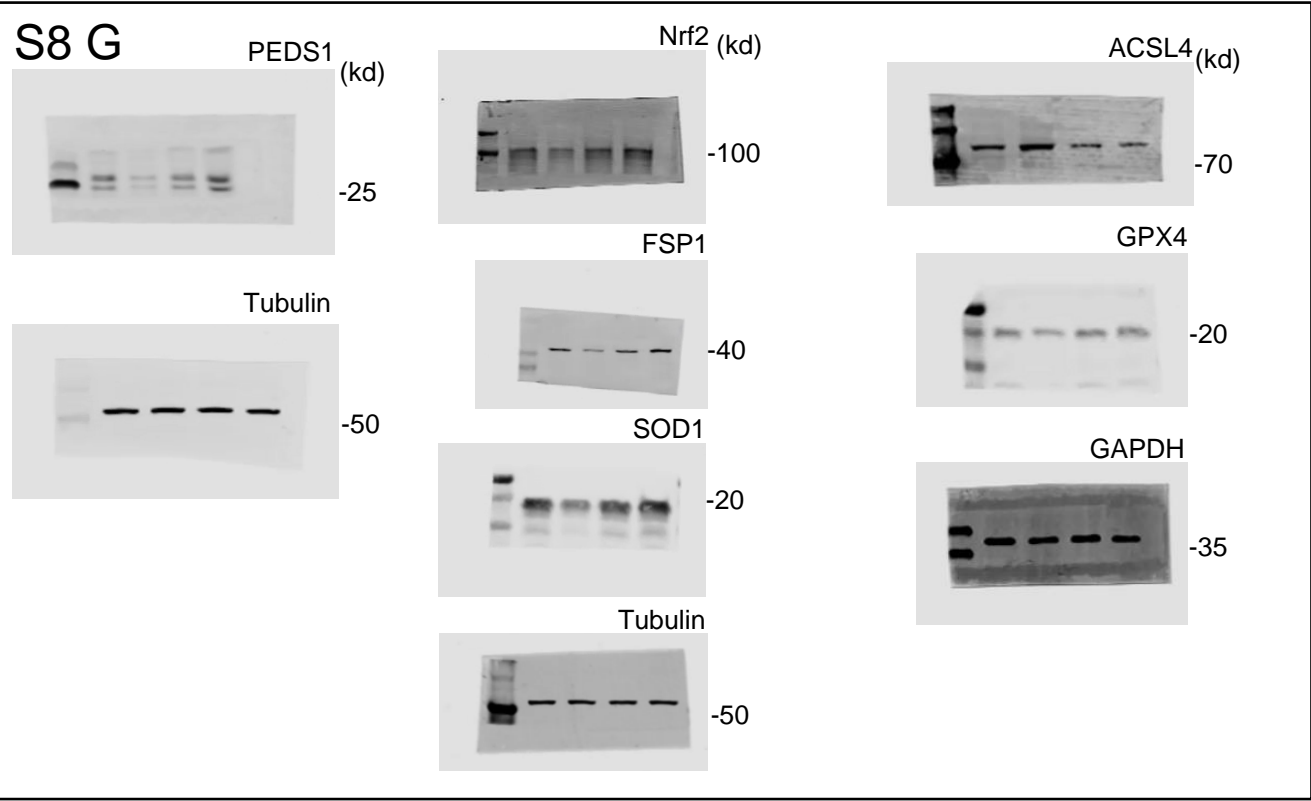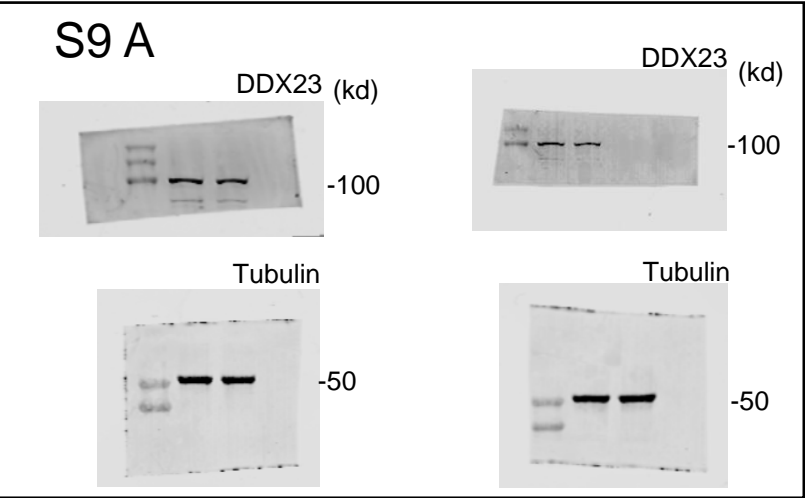

Supplement: Supplementary file 6 — Original Western Blots [file 41419_2025_8293_MOESM6_ESM.pdf]
